# Gas Fermentation—A Flexible Platform for Commercial Scale Production of Low-Carbon-Fuels and Chemicals from Waste and Renewable Feedstocks

**DOI:** 10.3389/fmicb.2016.00694

**Published:** 2016-05-11

**Authors:** FungMin Liew, Michael E. Martin, Ryan C. Tappel, Björn D. Heijstra, Christophe Mihalcea, Michael Köpke

**Affiliations:** LanzaTech, Inc.Skokie, IL, USA

**Keywords:** gas fermentation, acetogens, *Clostridium*, syngas, synthetic biology, coupled processes, carbon capture and utilization, low-carbon fuels

## Abstract

There is an immediate need to drastically reduce the emissions associated with global fossil fuel consumption in order to limit climate change. However, carbon-based materials, chemicals, and transportation fuels are predominantly made from fossil sources and currently there is no alternative source available to adequately displace them. Gas-fermenting microorganisms that fix carbon dioxide (CO_2_) and carbon monoxide (CO) can break this dependence as they are capable of converting gaseous carbon to fuels and chemicals. As such, the technology can utilize a wide range of feedstocks including gasified organic matter of any sort (e.g., municipal solid waste, industrial waste, biomass, and agricultural waste residues) or industrial off-gases (e.g., from steel mills or processing plants). Gas fermentation has matured to the point that large-scale production of ethanol from gas has been demonstrated by two companies. This review gives an overview of the gas fermentation process, focusing specifically on anaerobic acetogens. Applications of synthetic biology and coupling gas fermentation to additional processes are discussed in detail. Both of these strategies, demonstrated at bench-scale, have abundant potential to rapidly expand the commercial product spectrum of gas fermentation and further improve efficiencies and yields.

## Gas fermentation overview

### Introduction

In December 2015, 195 countries adopted the Paris Agreement at the end of the 21st Conference of the Parties to the United Nations Framework Convention on Climate Change. The agreement “aims to strengthen the global response to the threat of climate change” and seeks to hold the increase in global average temperature to “well below 2°C above pre-industrial levels” (United Nations, 2015). Though non-binding, this consensus underscores the rising urgency for actions that will limit the amount of greenhouse gasses emitted into the atmosphere. To achieve the goal of staying within the above mentioned 2°C target may require leaving a third of oil reserves, half of gas reserves, and over 80% of current coal reserves unused until 2050 (Friedlingstein et al., 2014; McGlade and Ekins, 2015). However, this time scale is likely too short to switch away from and eliminate the need for carbon-based transportation fuels and chemicals. To meet the demand for these products while simultaneously reducing greenhouse gas emissions, low-carbon fuels (i.e., fuels that emit less CO_2_ over their life cycle of production and use relative to fossil fuels) and chemicals are needed.

One renewable, non-fossil derived source for low-carbon fuels and chemicals is biomass. Plant biomass is the fourth largest renewable energy resource in the world, following geothermal, solar, and wind (Metz et al., 2007). It accounted for approximately 10% of the global energy supply in 2009 (Vakkilainen et al., 2013). Though the vast majority of this energy was used for inefficient residential heating and cooking, biomass is increasingly being converted to biofuels in efforts to displace fossil fuel-derived transportation fuel. However, only 3.5% of global oil demand for road transport (adjusted for energy content) was met by biofuels in 2013 (International Energy Agency, 2014). In order to successfully displace enough oil and gas reserves to mitigate climate change, significantly more biomass would need to be converted to fuels.

In addition to commercial ethanol production by yeast fermentation of starch/sugar from corn/sugar cane, low-carbon fuels can be produced from lignocellulosic biomass (Naik et al., 2010). There are various proposed process flows but generally multiple pretreatment and hydrolysis steps are necessary to separate cellulose and hemicellulose from the recalcitrant lignin and to break down polysaccharides into fermentable monosaccharides (Geddes et al., 2011). Steps are diverse and include steam explosion, ammonia fiber expansion (AFEX), extrusion, ionic liquid extraction, and dilute acid and enzymatic hydrolysis (Geddes et al., 2011; Brown and Brown, 2013). However, these methods are considered expensive (both in terms of cost and water usage), and no single pretreatment is universally effective (Haghighi Mood et al., 2013). Further challenges include separation of hexose (C6) and pentose (C5) sugars or isolating/designing a robust biocatalyst that can ferment both (Sánchez and Cardona, 2008; Geddes et al., 2011). An alternative to separating the pretreatment/hydrolysis and fermentation is to use cellulolytic microorganisms that are capable of performing both the hydrolysis of lignocellulosic material and sugar fermentation (termed “consolidated bioprocessing process,” or “CBP”). This has been demonstrated in laboratory conditions but not under industrial settings and challenges around conversion rates and productivities remain (Brown and Brown, 2013).

### Advantages of gas fermentation

Even with the extensive biomass processing required to produce cellulosic ethanol, the lignin, which can account for up to 40% of plant biomass does not get converted (Sun and Cheng, 2002; Abubackar et al., 2011). The use of the biomass in its entirety as a feedstock is a key advantage inherent to gas fermentation compared to sugar and cellulosic fermentation to produce low-carbon fuels. Biomass can be gasified to a mixture of carbon monoxide (CO), carbon dioxide (CO_2_), hydrogen (H_2_), and nitrogen (N_2_), also called synthesis gas or syngas. Conversion of biomass to syngas allows for utilization of nearly all the available carbon contained within the biomass, including the otherwise inaccessible lignin fraction, and bypasses the expense and inefficiencies of biomass pretreatment.

Once converted into a gas, there are two options for conversion to useful products. Traditionally this has been achieved using the Fischer-Tropsch process (FTP), but the technology has some drawbacks and is very capital intensive. The ability to fix gaseous, inorganic carbon into organic material (autotrophy) is also a prerequisite for life, and routes exist in various forms across all domains of life (Thauer, 2007). Eukaryotes (the most common example being photosynthesis in plants), archaea, and bacteria can all fix carbon by reducing CO_2_ and/or CO. Anaerobic gas-fermenting bacteria, specifically acetogens, are the focus of this review due to the advantages they possess in low-carbon fuel/chemical production. Advantages of gas fermentation over traditional FTP conversion (Section Fischer Tropsch Process vs. Gas Fermentation), advantages in substrate diversity (Section Substrate Diversity), and advantages inherent in acetogens (Section Acetogens and Wood-Ljungdahl Pathway) are discussed in detail below.

#### Fischer tropsch process vs. gas fermentation

Converting syngas to low-carbon liquid hydrocarbons with short to medium/long chains has been traditionally achieved via FTP. First developed in 1925, FTP employs high temperature (150–350 °C), elevated pressures (30 bar), and heterogeneous catalysts such as cobalt, ruthenium, and iron (De Klerk et al., 2013). In comparison, gas fermentation takes place at 37 °C and atmospheric pressure, which presents significant energy and cost savings relative to FTP. FTP, unlike gas fermentation, also requires a fixed H_2_:CO ratio of ideally ~2:1 (De Klerk et al., 2013). However, syngas derived from biomass has typically a lower H_2_:CO ratio (van der Drift et al., 2001; Datar et al., 2004; Boerrigter and Rauch, 2005; Boateng et al., 2007; Piccolo and Bezzo, 2009; Zheng et al., 2016), often requiring an extra step of water-gas shift reaction (National Energy Technology Laboratory, 2013) at the expense of CO to adjust the H_2_:CO for FTP. Although chemical processes are generally considered faster than biological approaches, the latter allow near complete conversion efficiencies due to the irreversible nature of biological reactions (Klasson et al., 1991, 1992). Furthermore, the high enzymatic specificities of biological conversions also result in higher product selectivity with the formation of fewer by-products. Crucially, the biocatalysts are also less susceptible to poisoning by sulfur, chlorine, and tars than the inorganic catalysts (Michael et al., 2011; Mohammadi et al., 2011), which reduces the gas pre-treatment costs.

#### Substrate diversity

**Figure 1:**
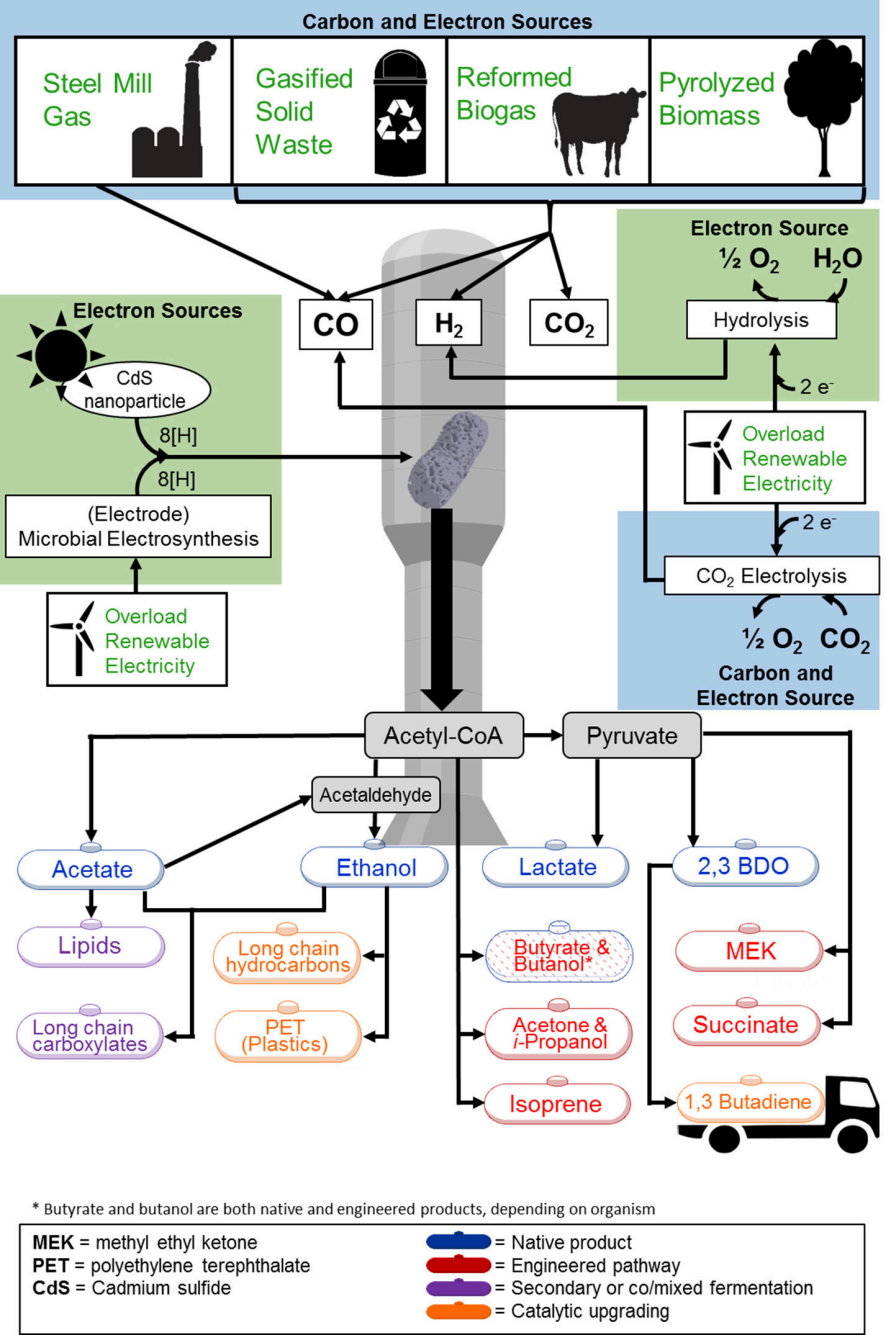
**Overview of feedstock and product options for gas fermentation**. Feedstocks to the gas fermentation platform are highlighted in light blue (carbon and electron sources) and green (electron sources). Feedstocks shown are at various stages of commercial deployment. Synthesis of all products shown has been demonstrated including (1) native products (blue text), (2) synthetic products produced through genetic modification (red text), (3) products generated through secondary fermentation of co/mixed cultures (purple text), and (4) products achieved through additional catalytic upgrading (orange text). Acronyms: 2,3-BDO, 2,3-Butanediol; MEK, methyl ethyl ketone.

With these capabilities (using gaseous carbon, flexible gas compositions, and tolerance to more contaminants over FTP), gas-fermenting microorganisms can make use of a diverse pool of substrates. Beyond biomass, other organic matter such as municipal solid waste (MSW), or organic industrial waste can be used as input for gasification (National Energy Technology Laboratory, 2014). Furthermore, off-gases from industrial processes, such as steel production (Clarke Energy, 2015) and reformed biogas (Oakley et al., 2011), can serve as direct substrates for gas fermentation (Figure 1). Gas fermentation, therefore, can increase the cyclical carbon emission and fixation in fossil fuel-consuming and carbon-emitting industries. Carbon containing off-gases produced in steel mills, for example, can be sequestered and converted into microbial biomass, fuels, and chemicals. Recycling carbon in this manner can decrease the need for tapping into fossil fuel reserves.

**Figure 2:**
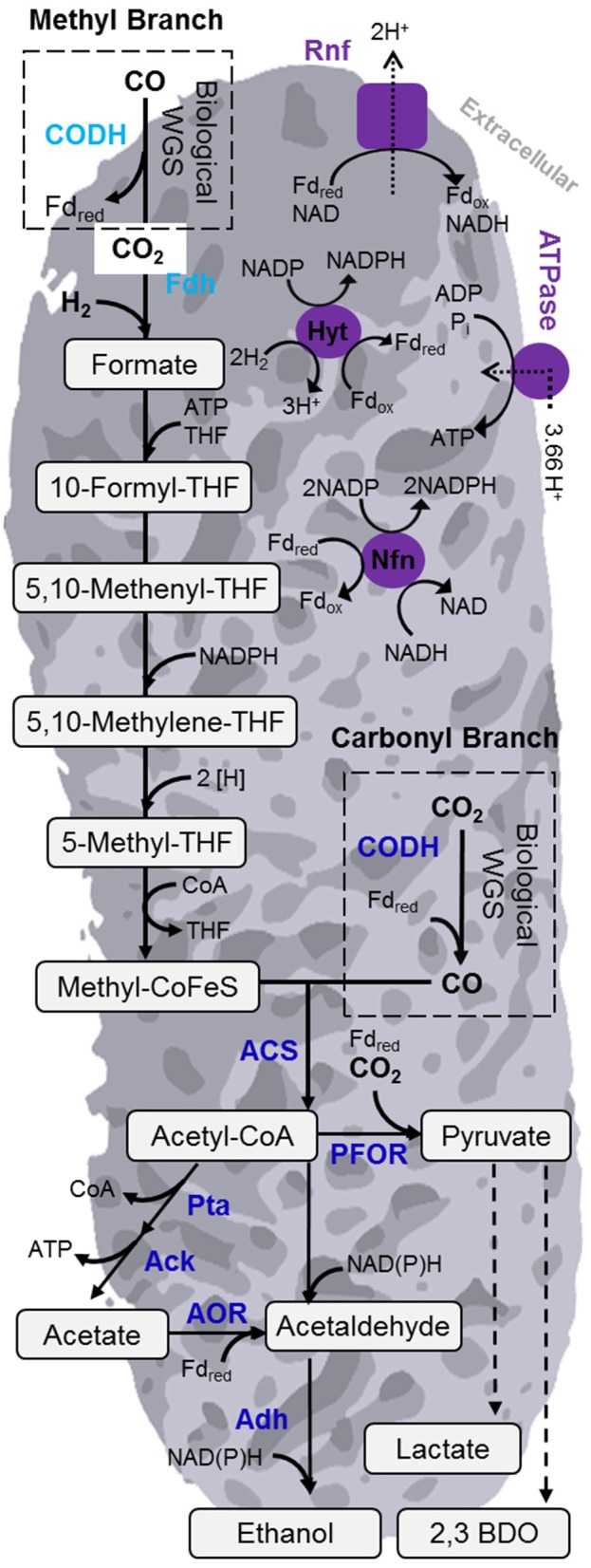
**Overview of Wood-Ljungdahl pathway (WLP) and energy conserving mechanisms of acetogen ***C. autoethanogenum*****. The WLP is central to the gas fermentation platform for carbon fixation. Noteworthy enzymes are in labeled in blue. The enzymes involved in energy conservation are shown in purple. Acronyms: 2,3-BDO, 2,3-butanediol; AOR, aldehyde:ferredoxin oxidoreductase; ACS, acetyl-CoA synthase; CODH, carbon monoxide dehydrogenase; Nfn, transhydrogenase; PFOR, pyruvate:ferredoxin oxidoreductase; Rnf, *Rhodocbacter* nitrogen fixation; THF, Tetrahydrofolate; WGS, water-gas shift reaction.

To fix the relatively oxidized carbon contained in these various syngas sources, acetogens (and other gas-fermenting microorganisms) require reducing equivalents in the form of electrons (such as NAD(P)H or reduced ferredoxin) to reduce the carbon to the central building block acetyl-CoA and further to reduced products such as alcohols. CO and H_2_ present in syngas themselves can provide these reducing equivalents (see Figure 2 and Section Acetogens and Wood-Ljungdahl Pathway below) by oxidation to CO_2_ and water (protons), respectively. Reducing equivalents can also be derived from sources other than the syngas sources discussed above.

One approach is to use electricity for water electrolysis (produces H_2_) and/or CO_2_ electrolysis (produces CO). If the electricity required for water or CO_2_ electrolysis can be provided by intermittent renewable sources (e.g., wind or solar, see Figure 1), it will not contribute to additional carbon emissions. This approach presents the potential to provide storage of excess electricity during times of low grid demand or surges. Storing excess energy in the form of low-carbon liquid fuels and chemicals is attractive as these chemical are energy dense and no loss of energy over time is observed as for conventional electricity storage.

Several technologies for electrolyzing water using renewable electricity to generate H_2_ and O_2_ have already been scaled up and are available commercially. Siemens utilizes a proton-exchange membrane in its Silyzer 200 product with reported overall efficiency of 65–70% (Siemens, 2013). Together with partners Stadtwerke Mainz, Linde and the RheinMain University of Applied Sciences they are operating the world's largest hydrogen electrolysis facility that will be able to process up to 6 mW of power (Aschenbrenner, 2016). Alternatively, Norsk Hydro's subsidiary, NEL Hydrogen, advertises a commercial-scale atmospheric electrolyser (NEL Hydrogen, 2015).

To date, the highest gas fermentation ethanol yields and selectivities have been demonstrated with CO-rich feedstocks (Gaddy et al., 2007), providing additional incentive to develop CO_2_ electrolysis technology. As mentioned before, it is also of note that during gas fermentation using CO only, a portion of the CO substrate pool must be converted to CO_2_ to provide necessary reducing equivalents for fixing the gaseous carbon. Some of this CO_2_ is not fixed and instead emitted from the cell, lowering the yield of carbon substrate fixed. CO_2_ electrolysis could be used to remedy this situation. Technologies to convert CO_2_ to CO are still at a pre-commercial stage. Examples include startups such as Opus12 (OPUS 12, 2015) and Dioxide Materials (Ritter, 2015), who report 99% CO_2_ conversion and greater than 80% efficiency using a proprietary membrane. Alternatively, researchers have found CO_2_ can be absorbed and reduced to CO using electricity and sponge-like crystals with a efficiency of 90% (Lin et al., 2015).

Another way of providing reducing equivalents is directly from electricity without a gaseous intermediary. Termed microbial electrosynthesis (MES), some acetogenic microorganisms have been shown to use electrical current to reduce CO_2_ to multi-carbon products (Nevin et al., 2010; Tremblay and Zhang, 2015; Figure 1). It was demonstrated that pure cultures of acetogens such as *Clostridium ljungdahlii, Clostridium aceticum, Moorella thermoacetica*, and *Sporomusa ovata* (but not the sodium dependent organism *Acetobacterium woodii*) form biofilms on the cathode surface of microbial fuels cells and while doing so consume current and generate acetate with 85% electron recovery (Nevin et al., 2011). Photosynthesis, by contrast, only offers between a 2.9 and 4.3% solar-to-biomass conversion efficiency for most crops (Zhang, 2015). MES is an efficient process but biological and engineering challenges remain regarding cultivation and maintenance of a dense biofilm, electron transfer at the cathode, and scale up.

A recent Science paper described an innovative method for direct electron input to the Wood-Ljungdahl pathway (WLP, described in the following section) of an acetogen by photosensitizing the microbes (Sakimoto et al., 2016; Figure 1). When grown with cadmium nitrate and cysteine, *M. thermoacetica* was able to use biologically generated cadmium sulfide (CdS) semiconducting nanoparticles which absorb light and use the energy to carry out photosynthesis. By feeding reducing equivalents directly into the WJP, solar energy can be converted into acetyl-CoA with 90% selectivity to acetate and 10% selectivity to biomass. This could be an efficient process to convert solar energy into liquid energy (i.e., fuels). However, it is in very early-stage development and requires biological optimization (e.g., production rates, solving issue of photo-oxidation of cell membranes, and selectivity toward other products), and engineering optimization (e.g., scale up and a reactor design where microbes are exposed to sufficient light or light/dark cycles).

#### Acetogens and wood-ljungdahl pathway

The advantages discussed above can be applied generally to autotrophic microorganisms. Acetogens are particularly attractive for commercialized gas fermentation due to their native ability to synthesize useful products such as ethanol, butanol and 2,3-butanediol and the fact that they are anaerobes. Anaerobic conditions avoid flammability issues working with combustible gases and also makes biological contamination less likely in a sugar and oxygen-free atmosphere. Acetogens are found in over 20 different genera and over 100 different species have been described (Imkamp and Müller, 2007). Table 1 provides an overview of the most noteworthy acetogenic species.

**Table 1:** **Overview of acetogens**.

**Organism**	**Substrates**	**Product(s)**	**T_opt_(°C)**	**pH_opt_**	**Genome sequence available**	**GEM available**	**References**
**MESOPHILIC MICROORGANISMS**							
*Acetobacterium woodii*	H_2_/CO_2_, CO	Acetate	30	6.8	Yes		Genthner and Bryant, 1987; Poehlein et al., 2012
*Acetonema longum*	H_2_/CO_2_	Acetate, butyrate	30–33	7.8	Draft		Kane and Breznak, 1991
*Alkalibaculum bacchi*	H_2_/CO_2_, CO	Acetate, ethanol	37	8.0–8.5			Allen et al., 2010; Liu et al., 2012
*Butyribacterium methylotrophicum*	H_2_/CO_2_, CO	Acetate, ethanol, butyrate, butanol	37	6			Zeikus et al., 1980; Lynd et al., 1982; Grethlein et al., 1991
*Clostridium aceticum*	H_2_/CO_2_, CO	Acetate	30	8.3	Yes		Adamse, 1980; Braun et al., 1981; Poehlein et al., 2015
*Clostridium autoethanogenum*	H_2_/CO_2_, CO	Acetate, ethanol, 2,3-butanediol, lactate	37	5.8–6.0	Yes	Yes	Abrini et al., 1994; Köpke et al., 2011; Brown et al., 2014; Marcellin et al., 2016
*Clostridium carboxidivorans* or “P7”	H_2_/CO_2_, CO	Acetate, ethanol, butyrate, butanol, lactate	38	6.2	Draft		Liou et al., 2005; Bruant et al., 2010
“*Clostridium coskatii”*	H_2_/CO_2_, CO	Acetate, ethanol	37	5.8–6.5			Zahn and Saxena, 2012
*Clostridium difficile*	H_2_/CO_2_, CO	Acetate, ethanol, butyrate	35–40	6.5–7.0	Yes		Rieu-Lesme et al., 1998; Monot et al., 2011; Köpke et al., 2013b
*Clostridium drakei*	H_2_/CO_2_, CO	Acetate, ethanol, butyrate	25–30	3.6–6.8	Draft		Küsel et al., 2000; Liou et al., 2005; Gössner et al., 2008; Jeong et al., 2014; Bengelsdorf et al., 2015
*Clostridium formicoaceticum*	CO	Acetate, formate	37	NR			Andreese et al., 1970; Diekert and Thauer, 1978; Lux and Drake, 1992
*Clostridium glycolicum*	H_2_/CO_2_	Acetate	37–40	7.0–7.5			Ohwaki and Hungate, 1977; Küsel et al., 2001
*Clostridium ljungdahlii*	H_2_/CO_2_, CO	Acetate, ethanol, 2,3-butanediol, lactate	37	6	Yes	Yes	Tanner et al., 1993; Köpke et al., 2010; Nagarajan et al., 2013
*Clostridium magnum*	H_2_/CO_2_	Acetate	30–32	7.0			Schink, 1984; Bomar et al., 1991
*Clostridium mayombei*	H_2_/CO_2_	Acetate	33	7.3			Kane et al., 1991
*Clostridium methoxybenzovorans*	H_2_/CO_2_	Acetate, formate	37	7.4			Mechichi et al., 1999
“*Clostridium ragsdalei” or “P11”*	H_2_/CO_2_, CO	Acetate, ethanol, 2,3-butanediol, lactate	37	6.3			Huhnke et al., 2008; Köpke et al., 2011
*Clostridium scatologenes*	H_2_/CO_2_, CO	Acetate, ethanol, butyrate	37–40	5.4–7.5			Küsel et al., 2000; Liou et al., 2005
*Eubacterium limosum*	H_2_/CO_2_, CO	Acetate, butyrate	38–39	7.0–7.2	Yes		Genthner et al., 1981; Genthner and Bryant, 1987; Jeong et al., 2015
*Oxobacter pfennigii*	H_2_/CO_2_, CO	Acetate, butyrate	36–38	7.3	Draft		Krumholz and Bryant, 1985
*Blautia productus*	H_2_/CO_2_, CO	Acetate	37	7			Lorowitz and Bryant, 1984
**THERMOPHILIC MICROORGANISMS**							
*Moorella thermoacetica*	H_2_/CO_2_, CO	Acetate	55	6.5–6.8	Yes	Yes	Kerby and Zeikus, 1983; Daniel et al., 1990; Pierce et al., 2008
*Moorella thermoautotrophica*	H_2_/CO_2_, CO	Acetate	58	6.1			Savage et al., 1987

*NR, Not reported; GEM, genome-scale network reconstruction*.

What makes the biology of acetogens particularly effective is the WLP for CO_2_ fixation. The WLP, also known as the reductive acetyl-CoA pathway, is the only linear CO_2_ fixation pathway to acetyl-CoA (Drake et al., 2008) and considered as the most efficient non-photosynthetic carbon fixation mechanism (Fast and Papoutsakis, 2012). There are several excellent reviews on the detailed mechanism and enzymes of the WLP (Wood, 1991; Drake et al., 2006; Ragsdale and Pierce, 2008; Latif et al., 2014). Briefly, the WLP consists of two branches, a methyl (Eastern) and a carbonyl (Western) branch (Figure 2). In the methyl branch, CO_2_ is reduced to formate. Next, the formate is activated by condensation with tetrahydrofolate (THF) to form formyl-THF, consuming one molecule of ATP. Over several reactions, formyl-THF is reduced to methyl-THF. In the final step of the methyl branch, the methyl group is transferred to a corrinoid iron-sulfur-containing protein (CoFeSP) and then fused to a molecule of CO from the carbonyl branch to form acetyl-CoA via the bifunctional carbon monoxide dehydrogenase/acetyl-CoA synthase (CODH/ACS) complex. When grown autotrophically on CO, the CO_2_ required for the methyl branch is generated by the CODH-catalyzed water-gas shift reaction. Likewise, during autotrophic growth on CO_2_, the CO is formed from CO_2_ by CODH in the carbonyl branch (Figure 2). The WLP is also active during heterotrophic growth, where released CO_2_ can be re-assimilated (Drake et al., 2006). This ability is also exploited in a concept called acetogenic or anaerobic, non-photosynthetic mixotrophy (ANP) to maximize yield in fermentation of sugar with additional hydrogen (Fast et al., 2015).

Even though the WLP is considered as extremely efficient (Fast and Papoutsakis, 2012), it still requires energy in form of one mol ATP per mol of acetyl-CoA formed. This can be balanced via substrate-level phosphorylation (SLP) during the acetate kinase reaction (Figure 2). However, this would require all acetyl-CoA formed in the WLP to be converted to acetate. Therefore, there must exist another mode of energy production to support cell growth and formation of other products. A membrane based mechanism for energy conservation has been proposed, but up to a few years ago exact details were not known, in particular for acetogens that lack cytochromes (Müller, 2003). This changed with the discovery of an Rnf complex in *A. woodii* (Müller et al., 2008). The Rnf complex (originally identified in *Rhodobacter* where it plays a role in nitrogen fixation) is able to build up a transmembrane electrochemical sodium ion gradient via coupling to an exergonic electron-transfer reaction. This gradient can then drive ATP synthesis by a membrane-bound F_1_F_*O*_ ATPase (Biegel and Müller, 2010; Biegel et al., 2011; Hess et al., 2016; Figure 2). Subsequently, the same mechanism (although proton rather than sodium ion dependent) was also identified in *C. ljungdahlii* (Köpke et al., 2010; Tremblay et al., 2012; Hess et al., 2016) and other acetogens such as *Clostridium autoethanogenum* (Mock et al., 2015) and *C. aceticum* (Poehlein et al., 2015). Not all acetogens possess such an Rnf complex. *M. thermoacetica*, for example, was found to contain cytochromes and a membrane-bound Ech (energy converting hydrogenase) complex to produce the necessary ion gradient (Huang et al., 2012).

Around the same time the role of the Rnf complex was elucidated, a novel energy conserving mechanism that involves flavin-based bifurcation of electrons was identified in anaerobes (Buckel and Thauer, 2013; Peters et al., 2016). The initial discovery came for a butyryl-CoA dehydrogenase in a non-acetogenic *Clostridium kluyveri* (Herrmann et al., 2008). Already, several electron bifurcating enzymes have been identified in acetogens since (Wang et al., 2010, 2013a,b; Schuchmann and Müller, 2012; Bertsch et al., 2013; Weghoff et al., 2014) and it is yet to be determined how prevalent this mechanism is (Peters et al., 2016). Important examples include hydrogenase complexes of *A. woodii, M. thermoacetica*, and *C. autoethanogenum* (Schuchmann and Müller, 2012; Wang et al., 2013a,b) or the NADH-dependent reduced ferredoxin:NADP oxidoreductase (Nfn) of *C. autoethanogenum* (Wang et al., 2010, 2013a; Figure 2).

While these unique mechanisms for energy conservation are widespread in acetogens, they are not found in traditional model organisms like *E. coli* or yeast. The specific differences in energy metabolism between the various acetogens are described in detail elsewhere (Schuchmann and Müller, 2014; Bertsch and Müller, 2015; Diender et al., 2015; Poehlein et al., 2015). Along with some genetic variations these mechanisms directly dictate the observed product spectrums. As an example, *A. woodii, C. aceticum*, and *M. thermoacetica* are only producing acetate under autotrophic conditions, despite having the genetic outfit for ethanol production and being able to produce ethanol under heterotrophic conditions growing on sugars. By contrast, *C. autoethanogenum, C. ljungdahlii*, “*Clostridium ragsdalei,” “Clostridium coskatii”* and *C. carboxidivorans* are able to synthesize more reduced products such as ethanol, butanol or 2,3-butanediol under autotrophic conditions. While the WLP genes and enzymes are highly conserved between all these organisms (Poehlein et al., 2015), they differ in some key enzymes such as presence of an electron-bifurcating hydrogenase, electron bifurcating Nfn, and an aldehyde:ferredoxin oxidoreductase (AOR) that enable formation of these more reduced products (Mock et al., 2015).

For commercial applications, mainly *C. ljungdahlii* type strain PETC (ATCC 55383 = DSM13528) (Tanner et al., 1993) and later isolates ERI-2 (ATCC 55380) (Gaddy, 1997), C-01 (ATCC 55988) (Gaddy, 2000), and O-52 (ATCC 55989) (Gaddy, 2000); *C. autoethanogenum* type strain JAI-1 (DSM10061) (Abrini et al., 1994) and evolved versions LBS1560 (DSM19630) (Simpson et al., 2009b) and LBS1561 (DSM23693) (Heijstra et al., 2012); *C. carboxidivorans* type strain P7 (ATCC BAA-624 = DSM 15234) (Liou et al., 2005); and isolates “*C. ragsdalei*” (ATCC BAA-622) (Huhnke et al., 2008) and “*C. coskatii*” (Zahn and Saxena, 2012) are considered. The latter two isolates have only been described in patent literature and not been characterized in a systematic journal. It is worth noting the importance that adaptive laboratory evolution plays in developing the strains being employed for commercial projects. Production strains often need to be adapted for high ethanol production under different fermentation conditions. For example, the *C. autoethanogenum* type strain JAI-1 has been reported to have low biomass growth, poor ethanol production, and minimal 2,3-butanediol production (Cotter et al., 2009; Guo et al., 2010; Köpke et al., 2011; Abubackar et al., 2015b), but the evolved *C. autoethanogenum* strain LBS1561 has demonstrated much greater production rates of 195 g/L/d ethanol (Molitor et al., 2016) and 14 g/L/d 2,3-butanediol (Simpson et al., 2014). Highest reported ethanol production rate in the literature was 360 g/L/d with *C. ljungdahlii* strain C-01, but in this case elevated pressure (1.7-2 bar) rather than atmospheric pressure was used (Gaddy et al., 2007).

### Commercialization and life-cycle analysis

The efficiency, diversity of substrates, and product selectivity advantages of gas fermentation have led to scaling up the fermentation process for commercial-scale production of low-carbon fuels using acetogens. Three companies, Coskata, INEOS Bio, and LanzaTech have operated pilot and demonstration plants for extended periods of time. Coskata's technology formed the basis of a new company Synata Bio (Lane, 2016) but has not been scaled up further. INEOS Bio and LanzaTech, on the other hand, are currently scaling up their processes to commercial scale.

INEOS Bio built an 8 million gallon per year (Mgy) semi-commercial facility in Vero Beach, FL as a joint venture with New Planet Energy Holdings, LLC. Commissioned in 2012, the facility uses lignocellulosic biomass and MSW for generating gas fermentation substrates and generates 6 mW of electrical power. In July 2013, the company announced successful production of ethanol in its facility (Schill, 2013). In September 2014, operational changes were imposed to optimize the technology and de-bottleneck the plant to achieve full production capacity (INEOS Bio, 2014).

LanzaTech has successfully operated two 0.1 Mgy pre-commercial plants in different locations in China with two steel companies, BaoSteel and Shougang Steel. Both plants, the first at one of BaoSteel's mills in Shanghai in 2012 and the second at a Shougang steel mill near Beijing in 2013, used steel mill off-gases as substrates for gas fermentation. The Shougang facility was certified by SCS Global Services in 2013 according to Roundtable on Sustainable Biomaterials (RSB) principals (SCS Global Services, 2013). The RSB is a global sustainability standard and certification system for biofuels and biomaterials production. In 2015, both China Steel Corporation of Taiwan and ArcelorMittal of Luxembourg approved commercial projects with LanzaTech. The former will be a 17 Mgy facility with the intention to scale up to 34 Mgy (Lane, 2015b). The latter 9.8 Mgy facility will be built at ArcelorMittal's flagship steel plant in Ghent, Belgium with the intention to construct further plants across ArcelorMittal's operations (Lane, 2015c). If scaled up to its full potential in Europe, the technology could enable the production of around 104 Mgy which would displace 1.6 million barrels of fossil fuel-derived gasoline on a BTU basis. In addition to these two projects, Aemetis, Inc. acquired a license from LanzaTech for the conversion of agricultural waste, forest waste, dairy waste and construction and demolition waste (CDW) to ethanol in California. In a first phase, Aemetis plans to adopt the process by adding an 8 Mgy gas fermentation unit to its existing 60 Mgy first generation biofuel facility in Keyes. This technology enables Aemetis to produce advanced ethanol that is valued up to approximately $3 per gallon more than traditional ethanol (LanzaTech, 2016).

Though these are promising signs that low-carbon fuel production by gas fermentation may be a commercial success in the near future, ongoing analyses in the form of Life Cycle Assessments (LCA) are necessary to ensure that environmental concerns are fully evaluated before commercial deployment. In a recently published cradle-to-grave LCA by Handler et al. (2016), the production of ethanol from gas fermentation in the USA is estimated to result in 67% Greenhouse gas (GHG) reduction (using blast oxygen furnace off-gas from steel manufacturing) and 88–98% GHG reduction (utilizing gasified biomass), when compared to conventional fossil gasoline. In both feedstock scenarios, 20–40% of the carbon in feed-gas is converted into ethanol. Conclusions drawn from LCA are often highly geographically dependent. A separate and older study showed that approximately 50% GHG savings can be attained from a microbial gas-to-ethanol platform based in China, relative to fossil gasoline (Ou et al., 2013).

### Process overview

The overall gas fermentation process can be broadly divided into four steps: (1) accumulation or generation of syngas; (2) gas pretreatment; (3) gas fermentation in a bioreactor; and (4) product separation.

#### Generation of syngas

The versatility of acetogenic bacteria to ferment syngas of diverse compositions means virtually any carbonaceous materials can be gasified to generate the feedstock as discussed in Section Substrate Diversity. When gasification is utilized, the carbonaceous material reacts with steam and air at elevated temperature (600–1000 °C) (Griffin and Schultz, 2012) and high pressure (>30 bar) (Breault, 2010) to form syngas of variable composition (depending on input and process parameters). Although a small amount of energy input is required to heat the incoming feedstock to gasification temperature at the beginning of the process, at steady-state the process is self-sustaining (Handler et al., 2016). Excess heat generated from gasification can be used to generate steam for product distillation and/or electricity (Handler et al., 2016). Depending on the type of gasifier, the starting material may be subjected to drying, commination (size reduction), chipping, pelletization, torrefaction, pyrolysis and/or pulverization prior to gasification (Bronson et al., 2012; Isaksson et al., 2013). Defined by how the reactor brings about contact with the feedstock and reactive gas, there are four main gasifier configurations: fixed/moving bed, fluidized bed, entrained flow, and transport flow (McKendry, 2002; Breault, 2010). Fluidized bed gasifiers are currently the most commonly used biomass gasifiers due to their ease of up-scaling, isothermal operation conditions and high feedstock conversion efficiencies (Swanson et al., 2010; Siedlecki et al., 2011; Griffin and Schultz, 2012).

#### Gas pretreatment

In addition to the main constituents CO, H_2_, CO_2_, input gas streams can also contain impurities such as particulates, tar, aromatics grouped as benzene, toluene, ethylene, xylenes (BTEX) and naphthalene, sulfur compounds such as hydrogen sulfide (H_2_S), carbonyl sulfide (COS), and carbon disulfide (CS_2_), halogens such as chlorine and hydrogen fluoride (HF), and other potentially inhibiting gases such as ammonia (NH_3_), nitric oxide and nitrogen dioxide (NO_x_), acetylene, oxygen (O_2_), reactive oxygen species (ROS), and hydrogen cyanide (HCN). These are generated for example during the gasification process, pyrolysis or manufacturing and can be present in fluctuating quantities (Oremland and Taylor, 1975; Abubackar et al., 2011; Griffin and Schultz, 2012; Munasinghe and Khanal, 2012; Zahn, 2015). A complete understanding of impurity species, their concentration fluctuations based on syngas input, process variables as well as installed treatment capacity is critical to maintain optimal productivity. In addition, monitoring impurity accumulation patterns within the fermentation is required to determine biological tolerance levels and the minimal inhibitory concentration (MIC) for designing economical treatment capacity. Understanding the effect of impurities could save treatment costs, however failure to do so can cause delays reaching full scale production capacity as shown in one large-scale syngas fermentation endeavor (Lane, 2014).

Even with gas-fermenting microorganisms' abilities to grow in the presence of low levels of impurities, some impurities necessitate near complete removal from an operational, biological and/or product specificity perspective. Particulates can be removed by cyclone separators and filters. Tars can be condensed and removed by quenching hot syngas, or, alternatively, can be reformed by heating at 800–900°C using olivine, dolomite, and nickel compounds as catalysts, generating additional syngas (McKendry, 2002).

Many contaminants including BTEX are lipophilic compounds that readily dissolve in the cytoplasmic membrane affecting membrane fluidity (Sikkema et al., 1995). Although polycyclic aromatics do not readily dissolve in aqueous phase, they can accumulate and negatively affect operations. Removal technologies for aromatics from gas are commercially available, and techniques to improve efficiency are still being reported (Ye and Ariya, 2015). O_2_'s toxicity above microoxic levels is particularly critical during inoculation of a bioreactor, when little biomass has accumulated to withstand introduction of aerobic conditions. O_2_ can be tolerated in certain microoxic conditions (Kawasaki et al., 2005). *C. ljungdahlii* has been shown to detoxify O_2_ and ROS (likely via rubrerythrin and hydrogen peroxidases) and ethanol formation could actually be stimulated by exposure to O_2_ (likely due to changes in AOR activity and co-factor metabolism) (Whitham et al., 2015). O_2_ can be removed by passing the gas over various metal catalysts such as Pt, Pd, and Cu (Yan et al., 2013). Using biological co-culture for O_2_ removal has also been described (Wu et al., 2016). Sulfur-containing impurities (e.g., H_2_S and COS) can poison the metal based catalysts (Vega et al., 1990; Rodriguez and Hrbek, 1999) and require prior removal despite the microorganisms' ability to grow in their presence (Griffin and Schultz, 2012).

Acetylene, NO_x_ and HCN are considered particularly troublesome as they are known to inhibit enzymes responsible for initial harvesting of energy from syngas (Anderson et al., 1993; Shima and Ataka, 2011). Cyanide binds to CODH (Ragsdale et al., 1983), a key enzyme of the WLP. NO is a non-competitive inhibitor of hydrogenase activity (Ahmed and Lewis, 2007) while acetylene reversibly inhibits hydrogenases (Krasna and Rittenberg, 1954; He et al., 1989; Maness and Weaver, 2001), which reduce ferredoxin for use in redox reactions (Figure 2). INEOS Bio has identified and reported HCN as a key contaminant that needs treatment from operation of their Vero Beach plant (INEOS Bio, 2014).

#### Gas fermentation

Treated syngas is next cooled and compressed then sparged into a bioreactor containing the gas-fermenting microorganisms in an aqueous medium. There are a multitude of variables to account for during gas fermentation. Bioreactor design, agitation, gas composition and supply rate, pH, temperature, headspace pressure, oxidation-reduction potential (ORP), nutrients, and amount of foaming in the bioreactor all can contribute to the goal of improving selectivity and yield of the desired product (e.g., ethanol and butanol) as discussed below.

One major obstacle immediately present in gas fermentation is the low solubility of the gaseous substrates and combined with an efficient transfer of their masses into the liquid media. CO, H_2_, and CO_2_ are soluble to approximately 28 mg/L, 1.6 mg/L, and 1.7 g/L (293 K, 1 atm), respectively, compared to 900 g/L for glucose, a prevalent substrate for traditional fermentations. As gas-fermenting microorganisms consume the gas, substrate availability can become rate-limiting. Increasing flow of the substrate gas can lead to decreased yields of product per mole of carbon fed to the reactor, making reactor design and operation crucial. Continuous stirred tank reactors (CSTR) offer excellent mixing and homogenous distribution of gas substrates to the microorganisms and are most commonly employed at laboratory scale (Ungerman and Heindel, 2007). However, the high power per unit volume required to drive the stirrer renders commercial scale operation economically challenging. Therefore, other less energy-demanding bioreactor designs such as bubble column, loop, and immobilized cell columns and their specific volumetric mass-transfer coefficients (k_L_a) that describes the efficiency of which a gas can be delivered to a bioreactor have been investigated intensively and reviewed elsewhere (Klasson et al., 1991; Bredwell et al., 1999; Ungerman and Heindel, 2007; Munasinghe and Khanal, 2010; Abubackar et al., 2011; Liew et al., 2013; Orgill et al., 2013).

Next to gas availability determined by reactor k_L_a, the ratio and partial pressures of CO, H_2_, and CO_2_ also influence the product yield, production rate, and selectivity of a gas fermentation (Genthner and Bryant, 1982; Vega et al., 1988; Klasson et al., 1991; Gaddy and Chen, 1998; Hurst and Lewis, 2010; Kantzow et al., 2015). CO and H_2_ are sources of electrons/reducing equivalents for reducing CO_2_ in the WLP and generating reduced products over acid products (e.g., ethanol vs. acetate). This product profile reflects the organism's requirement to maintain an internal energy balance that favors growth and is directly influenced by the gas composition and availability. As an example, productivity with *C. ljungdahlii* was improved from 38.4 g/L/d at 1 atmosphere to 360 g/L/d at 6 atmospheres (Gaddy et al., 2007). Another strategy to control the product profile is lowering the pH of the fermentation culture. This pH change can lead to a (reversible) shift from acidogenesis to solventogenesis allowing an increased production of ethanol and other highly reduced products (Grethlein et al., 1990; Gaddy and Claussen, 1992; Phillips et al., 1993; Guo et al., 2010; Abubackar et al., 2012, 2015b, 2016a,b; Richter et al., 2013b; Martin et al., 2016).

Besides gas, medium composition also affects product yield and selectivity. Nutrient optimization has proven to be a process and species specific requirement. Media optimizations have been conducted for many acetogens including *C. autoethanogenum* (Cotter et al., 2009; Guo et al., 2010; Simpson et al., 2010; Abubackar et al., 2015a, 2016a), *C. ljungdahlii* (Phillips et al., 1993), “*C. ragsdalei”* (Babu et al., 2010; Kundiyana et al., 2010, 2011; Panneerselvam et al., 2010; Maddipati et al., 2011; Phillips et al., 2011; Saxena and Tanner, 2011, 2012), *C. aceticum* (Sim and Kamaruddin, 2008), and *M. thermoacetica* (Lundie and Drake, 1984; Savage and Drake, 1986). B vitamins and metals such as zinc, nickel, selenium, and tungsten, required as cofactors for certain enzymes in the central metabolism, are required for bacterial growth and affect product selectivity. For an industrial process it is critically important to keep media cost low, for example by eliminating yeast extract requirements, recycling nutrients and usage of industrial-grade bulk chemicals.

#### Product separation

Finally, product separation is required to separate the desired metabolic product from the fermentation broth. Distillation systems are common to separate lower boiling point products such as ethanol (Handler et al., 2016) and acetone, but this is considered energy-intensive (and therefore potentially expensive), especially for low concentration products and products with high boiling points (e.g., butanediol). Other technologies to separate fermentation products from broth include liquid-liquid extraction, gas stripping, adsorption, perstraction, pervaporation, and vacuum distillation (Huang et al., 2008; Frolkova and Raeva, 2010; Liew et al., 2013; Molitor et al., 2016) Each of these separation technologies has their own benefits and drawbacks, including potential fouling of membranes (perstraction and pervaporation) and substrate removal (gas stripping and liquid-liquid extraction). Liquid-liquid extraction is also an option for removing acetate from the fermentation broth of gas-fermenting acetogens (Jipa et al., 2009).

## Synthetic biology approaches to expand product spectrum of gas fermentation

### Overview of synthetic biology for acetogens

Synthetic biology and metabolic engineering approaches offer great promise to improve the efficiency of gas fermentation and to expand the product spectrum beyond native products ethanol, acetate and butanediol to a range of higher-value fuels and commodity chemicals (Latif et al., 2014; National Research Council, 2015; Figure 1). Of particular interest are products that can be separated using the same distillation technology used for ethanol in current commercial endeavors. In these cases, only minimal modifications to the process conditions or fermentation regime would be required but not to the existing plant itself to shift from one product to another. This is a real paradigm shift to typical chemical production plants that cannot react to changing market conditions.

To approach these goals in acetogens, consider the model organisms, *E. coli* and yeast. These have successfully been reprogrammed to convert sugars at commercially relevant rates and concentrations to new products like farnesene, isobutanol, 1,3-propanediol (1,3-PDO), and 1,4-butanediol (1,4-BDO) (Lan and Liao, 2013; Cho et al., 2014; George et al., 2015; Burk and Van Dien, 2016). Although these advancements cannot be directly transferred to acetogens, similar principles may be applied. Historically, development has been hampered by a lack of available information and tools. *E. coli* and yeast are well studied and characterized on molecular and systems level with decades of intensive research by thousands of research groups. This has led to the development of sophisticated genetic toolkits that allow precise and rapid design (Temme et al., 2012b) and automated high-throughput strain engineering and prototyping (Gardner, 2013; Burk and Van Dien, 2016). In contrast, acetogens have been viewed as genetically inaccessible until a few years ago and are still considered difficult to work with (Burk and Van Dien, 2016). More specifically, cultivation under an industrial setting, the limited number of genetic tools (Burk and Van Dien, 2016), potential energetic constraints (Bertsch and Müller, 2015), and lack of characterization on genetic and molecular levels are considered key challenges to address for successful development of genetically engineered gas-fermenting platforms.

However, over the last 5 years, great strides have been made that have contributed to the understanding of acetogens on a molecular and systems level. These include elucidation of whole genome sequences for a range of acetogens including *C. autoethanogenum* (Brown et al., 2014; Humphreys et al., 2015; Utturkar et al., 2015), *C. ljungdahlii* (Köpke et al., 2010), *C. aceticum* (Poehlein et al., 2015), *M. thermoacetica* (Pierce et al., 2008), *A. woodii* (Poehlein et al., 2012), and *E. limosum* (Roh et al., 2011); identification of cofactors for key reactions for *C. autoethanogenum* (Wang et al., 2013a; Mock et al., 2015), *A. woodii* (Schuchmann and Müller, 2012, 2014), *M. thermoacetica* (Huang et al., 2012; Mock et al., 2014), and *E. limosum* (Jeong et al., 2015); and transcriptomics analyses of *C. autoethanogenum* (Mock et al., 2015; Marcellin et al., 2016) and *C. ljungdahlii* (Nagarajan et al., 2013; Tan et al., 2013; Whitham et al., 2015). Even more recently, a first complete systems level study comprising of transcriptome, metabolome, and proteome analyses of *C. autoethanogenum* was published (Marcellin et al., 2016). This study demonstrated that the ATP pool remains constant during autotrophic growth on gas and heterotrophic growth on fructose and dissected the underlying mechanisms. Additionally, a genome-scale model (GEM) was provided to allow more rational strain design. Similar models also exist for *C. ljungdahlii* (Nagarajan et al., 2013) and *M. thermoacetica* (Islam et al., 2015). Based on the *C. ljungdahlii* GEM, spatiotemporal modeling of gas fermentation in a bubble column was performed recently (Chen et al., 2015, 2016). With continued advancement, these GEMs may enable development of efficient strategies and strain designs in a manner similar to that observed for *E. coli* and yeast.

In parallel, a range of genetic tools have been developed or adapted for gas-fermenting acetogens. Most of the work has focused on acetogenic clostridia, in part due to their properties as commercial production strains for gas-fermentation (see Section Acetogens and Wood-Ljungdahl Pathway) but also due to the presence of pre-existing tools for non-acetogenic clostridia studied for medical purposes (e.g., *Clostridium difficile* and *Clostridium botulinum*), or exploited for acetone/isopropanol-butanol-ethanol (ABE/IBE) fermentation (e.g., *Clostridium acetobutylicum* and *Clostridium beijerinckii*) and or their ability to degrade cellulosic material (e.g., *Clostridium cellulolyticum*). An overview of genetic tools is provided in Section Genetic Tools Development. In Section Successful Examples of Metabolic Engineering of Gas-Fermenting Organisms to Expand Product Portfolio, details of several successful examples of metabolic engineering of acetogenic organisms are discussed. These results have been possible due to the enhanced understanding of acetogens and genetic tools development. To accelerate development of new commercially relevant strains and processes, it will be important to further refine and improve the existing tools and models and increase throughput in strain design and prototyping.

### Genetic tools development

#### DNA transfer

The establishment of an efficient protocol to introduce foreign DNA into a microbial host is frequently one of the most challenging steps in any genetic engineering endeavor. This is especially true for Gram-positive bacteria such as clostridia and most other acetogens due to the physical barrier of the thick peptidoglycan cell wall and highly active restriction-modification systems (Pyne et al., 2014). As a result of the inability to transfer DNA, the advancement of genetic tools in clostridia has been severely hindered and lagged behind genetically accessible model organisms such as *E. coli* or yeast. Some of the well-studied, non-acetogenic clostridia, such as the ABE model organisms *C. acetobutylicum* (Mermelstein et al., 1992) and *C. beijerinckii* (Oultram et al., 1988), and important pathogens, such as *C. difficile* (Ackermann et al., 2001) and *C. botulinum* (Zhou and Johnson, 1993), have transformation protocols that were established as early as 1988. It is intriguing that *C. difficile* was recently found to also harbor the WLP and is able to grow autotrophically on H_2_ and CO_2_ (Köpke et al., 2013b). Because it is a human pathogen, *C. difficile* is not considered for industrial use, but studies conducted on the metabolism and development of genetic tools for this pathogen are of interest for acetogens. Methods for genetic manipulation in acetogens other than *C. difficile* were only beginning to emerge since 2010 (Köpke et al., 2010).

Due to its technical simplicity, higher reproducibility, scalability, greater transformation efficiency, and independence from donor species, electroporation is the most commonly explored method of transformation in microorganisms. Transfer of foreign DNA was successfully demonstrated for *C. ljungdahlii* (Köpke et al., 2010; Leang et al., 2013), *C. autoethanogenum* (Köpke and Liew, 2011), *C. aceticum* (Schiel-Bengelsdorf and Dürre, 2012), *A. woodii* (Straub et al., 2014), and *M. thermoacetica* (Kita et al., 2013). With optimizations, a transformation efficiency of up to 1.7 × 10^4^ cfu/μg DNA for *C. ljungdahlii* was achieved by Leang et al. (2013). Advanced next generation sequencing platforms such as Single Molecule Real-Time (SMRT) sequencing can be employed to reliably detect certain methylation signatures (Flusberg et al., 2010; Fang et al., 2012) as it has been done for *C. autoethanogenum* (Utturkar et al., 2015). This information can then be used to formulate *in vitro* or *in vivo* methylation strategies or disruption of endonucleases to protect foreign DNA from the host's restriction barrier. For instance, genetic disruption of restriction endonuclease in non-acetogen *C. acetobutylicum* (Dong et al., 2010) and *C. cellulolyticum* (Cui et al., 2012) resulted in significantly improved transformation efficiency using unmethylated plasmids.

Alternatively, the transfer of foreign DNA into host cells can be accomplished via conjugation. This method requires direct cell-to-cell contact during the transfer of DNA from donor to recipient so this approach is limited by the host range of the donor. However, transfer of DNA via conjugation has been reported to occur in single strand, by which means the incoming DNA can evade the recipient's restriction endonucleases (Jennert et al., 2000; Purdy et al., 2002). Conjugation was reported for *C. autoethanogenum* (using *E. coli* strain HB101 as donor) (Mock et al., 2015), and *A. woodii* (using *E. coli* strain S17-1 as donor) (Strätz et al., 1994).

The presence of active restriction modification systems (methyltransferases and nucleases) in acetogens, coupled with low transformation efficiencies, means routine molecular biology work such as plasmid purification and ligation cloning have to be performed first in a separate “preparation” host (e.g., *E. coli*) before introduction into acetogens. This necessitates the development of shuttle plasmids that contain replicon(s) and selection marker(s) that enable the propagation and selection of the plasmids in both acetogens and “preparation” host. The modular *Clostridium-E. coli* shuttle plasmids developed by Heap et al. (2009) contain customizable components such as different Gram-positive and Gram-negative replicons as well as the *traJ* gene (for conjugation), and it has been adopted for genetic manipulation work in several acetogens (Köpke and Liew, 2011; Leang et al., 2013; Ueki et al., 2014; Hoffmeister et al., 2016). Future development of high-efficiency transformation protocols, use of non-circularized DNA, direct cloning and intact plasmid purification directly from acetogens will be important to significantly improve the workflow, throughput and automation of recombinant acetogen constructions.

#### Knock-down of target genes via antisense RNA (asRNA)

When genetic tools development was still in its infancy and stable inactivation mutants were difficult to generate, knock-down of target genes via the actions of plasmid-delivered antisense RNA (asRNA) represented an alternative route for genetic manipulation. In cases where the target gene is essential to survival of the microorganism, knock-down is still an option over a complete knock-out. By applying asRNA, up to 90% reduction in gene expression together with high level of specificity were reported for clostridia (Desai and Papoutsakis, 1999; Tummala et al., 2003; Scotcher et al., 2005).

#### Intron-based gene inactivation

Some of the first reliable mutagenic tools for clostridia were based on intron-based gene inactivation. The approach utilizes the specificity of mobile group II intron Ll.*ltrB* from *Lactoccocus lactis* to propagate into the specified site via a RNA-mediated, retro-homing mechanism (Karberg et al., 2001). Heap et al. (2007) adapted such mobile elements for use in a wide spectrum of clostridia, and the technology is termed “ClosTron.” Without a selection marker, the screening effort necessary for the isolation of the desired integrant can be immense due to the variability in integration frequency between target sites. By incorporating a clostridial retro-transposition activating marker (RAM) based on the *ermB* gene of the *Enterococcus faecalis* plasmid pAMβ1, positive selection for the desired integration can be conveniently made by the bacteria's acquisition of erythromycin resistance (Heap et al., 2007, 2010; Kuehne and Minton, 2012). Thanks to these properties, this technique is reproducible and has been widely applied to various clostridia (Heap et al., 2007, 2010; Camiade et al., 2010) including acetogen *C. autoethanogenum* (Mock et al., 2015; Marcellin et al., 2016). The technique should also be amendable to other non-clostridia mesophilic acetogens such as *A. woodii* but may not be applicable for some of the thermophilic organisms such as *M. thermoacetica*. This technique was further optimized to generate multiple insertional mutations in the same strain by recycling of marker through the actions of FLP recombinase, and the delivery of cargo sequence of up to 1 kb was successfully demonstrated in *C. sporogenes* (Heap et al., 2010).

#### Stable genome insertion or deletion via homologous recombination

For stable genome insertion, homologous recombination is typically used. To facilitate this method, several counter-selectable markers to allow efficient screening for the rare second recombination event have been developed for clostridia including *upp* (encoding a uracil-phosphoribosyl-transferase) (Soucaille et al., 2008; Croux et al., 2016), *mazF* (*E. coli* toxin) (Al-Hinai et al., 2012a), *codA* (cytosine deaminase) (Cartman et al., 2012), and *pyrE* or *pyrF* (orotate phosphoribosyltransferase) (Ng et al., 2013). PyrF has also been shown to be functional in thermophilic acetogen *M. thermoacetica* (Kita et al., 2013). For acetogen *C. autoethanogenum*, two additional counter-selectable marker pheS^*^ (modified phenylalanine tRNA synthase) and *thiK* (thymidine kinase) have been developed (Walker et al., 2015).

As alternative approach to the use of a counter-selectable marker, it is also possible to rely on single crossover recombination facilitated by an antibiotic marker and then excise the marker. This can either be achieved using above mentioned FLP recombinase (Lee et al., 2016) or using the Cre-lox system (Sauer, 1987). This system utilizes a site-specific recombinase and lox recognition sites, to delete the vector backbone and allowed the recycling of an antibiotic resistance gene. Cre-Lox has been successfully demonstrated in the acetogen *C. ljungdahlii* (Ueki et al., 2014).

#### Precise genetic manipulation via allelic-exchange

Recently, a novel genetic tool that allows stable genome insertion via homologous recombination was developed. Termed Allele-Coupled Exchange (ACE), this approach does not employ a counter selective marker to select for the rare second recombination event. Instead, it utilizes the activation or inactivation of gene(s) that result in a selectable phenotype, and asymmetrical homology arms to direct the order of recombination events (Heap et al., 2012). For example, a promoterless primary/secondary *adh* from *C. beijerinckii* coupled to the *ermB* gene was introduced into the downstream region of the *thlA* gene in the genome of *C. acetobutylicum*. The native promoter of *thlA* drives the expression of *ermB* and *adh*, allowing selection for the second recombination event using erythromycin or acid-stable analog clarithromycin, and also creation of a recombinant that reduces acetone to the more desirable isopropanol (Heap et al., 2012). Remarkably, the whole genome of phage lambda (48.5 kb minus a 6 kb region) was successfully inserted into the genome of *C. acetobutylicum* in three successive steps using this genetic tool. This technique was also demonstrated in *C. difficile* and *C. sporogenes*, suggesting it is applicable to other *Clostridium* spp. including acetogens (Heap et al., 2012; Ng et al., 2013).

#### Precise genetic manipulation via triple cross

**Figure 3:**
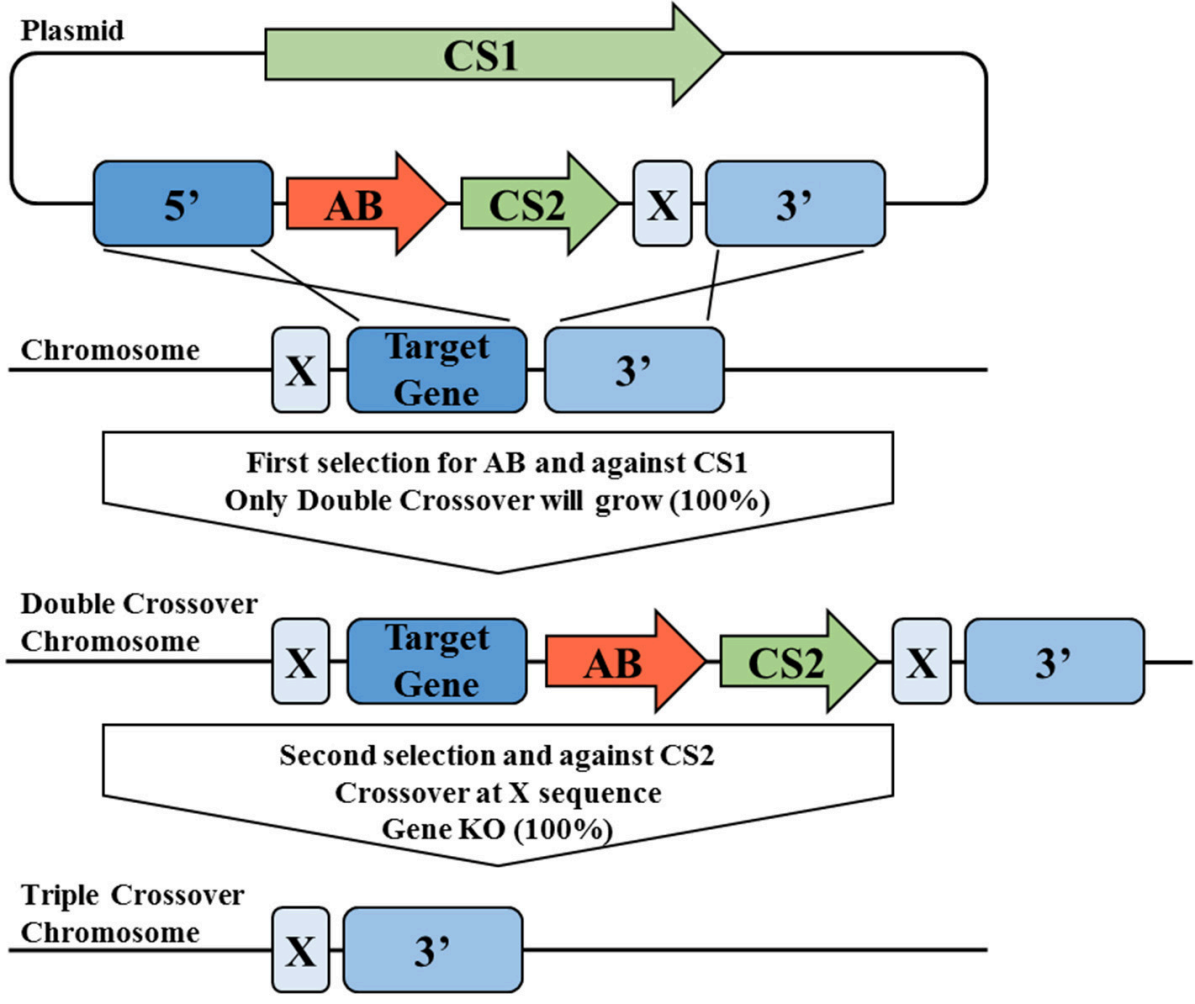
**Overview of the Triple Cross tool for precise genetic manipulations**. The triple cross tool relies on two counter-selectable markers (CS1 and CS2) in combination with one antibiotic marker (AB). CS1 and AB1 are located between two homology arms (5′ and 3′) together with a shorter, third homology arm (X). Using selection for AB and against CS1, a direct double crossover event at homology arms 3′ and 5′ is forced in a first step, this is facilitated by having homology arms of different length. In an optional second step, the marker can be recycled using shorter third homology arm X and selection against CS2. Shown is deletion of a target gene, but the same technology can also be used to deliver genes by placing the respective sequence between homology arms X and 3′. Depending how the homology arms are placed, the gene can either be inserted at any given position in the genome or an existing sequence be replaced by a new one.

Recently a novel allelic-exchange based tool termed “Triple Cross” for acetogen *C. autoethanogenum* was described in a patent application (Walker and Köpke, 2015). Unlike the classical two-step, double-crossover approach which involves one positive and one negative selection markers with two homology arms, the two-step Triple Cross tool utilizes one positive and two negative selection markers with three homology arms. Instead of screening for a first crossover in the first step and a second crossover with a marker recycling in the second step, this invention forces a double crossover directly in the first step using a combination of positive selection marker and a negative selection marker. The optional second step involves the recycling of selection marker through the third homology arms and the second negative selection marker in a third crossover event (Figure 3). Using this technique, a 2,3-butanediol dehydrogenase gene and a secondary alcohol dehydrogenase gene of *C. autoethanogenum* were successfully in-frame deleted in a “scarless”-manner (Walker and Köpke, 2015). This approach can also be extended to perform other forms of precise genetic manipulation, including insertion and point mutations.

#### Transposon mutagenesis

The genetic tools described so far constitute “reverse genetics,” in which a defined gene(s) is selectively inactivated or introduced to study the phenotype. In contrast, “forward genetics” do not make any assumptions about the genes involved and aim to determine the genotype resulting in a particular phenotype. To achieve this, a pool of random mutants needs to be generated. This can be accomplished by means of transposon mutagenesis. Earlier efforts of transposon mutagenesis were demonstrated in *C. saccharobutylicum* (Keis et al., 2001), *C. acetobutylicum*, and *C. beijerinckii*, but issues with multiple transposon insertions per mutant, and non-random distribution of insertions were also reported (Bertram and Dürre, 1989; Woolley et al., 1989). Recent developments have seen the successful generation of mono-copy random insertion of transposon *Tn1545* into *C. cellulolyticum* (Blouzard et al., 2010) and mariner transposon *Himar1* into *C. difficile* (Cartman and Minton, 2010; Zhang et al., 2015b). When coupled with next generation sequencing, transposon-directed insertion site sequencing (TraDIS) was performed on *C. difficile* and identified 404 essential genes for growth and 798 genes that are likely to affect spore production (Dembek et al., 2015). Recently, an inducible transposon system for random mutagenesis has been described for *C. acetobutylicum* that showed over 90% successful transposition (Zhang et al., 2016).

#### CRISPR/Cas9

The Clustered Regularly Interspaced Short Palindromic Repeats (CRISPR)/CRISPR-associated protein 9 (Cas9) system is a RNA-guided prokaryotic immune system which can cleave foreign DNA (e.g., phages and plasmids). The type II CRISPR/Cas9 system has been exploited for high-efficiency gene editing in model microorganisms such as *E. coli* (Jiang et al., 2015), *S. cerevisiae* (DiCarlo et al., 2013), and *Streptococcus pneumoniae* (Jiang et al., 2013). Recently, the CRISPR/Cas9 system from *Streptococcus pyogenes* was successfully applied to various clostridia (Wang et al., 2015; Xu et al., 2015) to deliver one-step, markerless, and highly efficient gene editing. For instance, DNA cargo of sizes 0.7 and 1.7 kbp (but not 3 and 6 kbp) were specifically inserted into the genome of *C. cellulolyticum* in a single step without marker using CRISPR/Cas9 nickase with single-nick triggered homologous recombination (Xu et al., 2015). This demonstrates potential in making use of this new technology in gas-fermenting microorganisms. Some acetogens as *C. autoethanogenum* also have a native CRSIPR system, that has been shown to be actively transcribed (Brown et al., 2014).

#### Genetic parts

In addition to the further development of tools, more validated parts are required to allow efficient metabolic engineering and refinement of the existing tools. This include variable-strength promoters, inducible expression systems, genetic circuits, transcription terminators, and ribosomal bindings sites (RBSs). For promoter library assembly, transcriptomic studies using RNA-seq can be used to identify promoters of various transcription intensities (Sharma and Vogel, 2014; Thomason et al., 2015).

Large libraries of promoters (Temme et al., 2012a), terminators (Chen et al., 2013b), and RBS calculators (Farasat et al., 2014) have been developed and characterized for model organisms like *E. coli*, dramatically increasing the control of metabolic pathways and allow efficient pathway refactoring and design of genetic circuits (Temme et al., 2012a,b). Though these parts may be transferable to acetogens, proper characterization/confirmation has not been completed.

There has been some progress on parts specifically from and for acetogens, specifically inducible promoter systems. A lactose-inducible system (*bgaR*-P_*bgaL*_) from *Clostridium perfringens* was successfully adapted for use in *C. ljungdahlii* (Banerjee et al., 2014) and a tetracycline inducible system (Tet3no) has been demonstrated in *C. autoethanogenum* (Walker and Köpke, 2015).

To make full use of these and any new genetic parts generated for acetogens in the future, reporter genes will need to be employed to assess the expression level and localization of a gene product. However, commonly used reporters such as green fluorescent protein (GFP) require oxygen for the development of the chromophore responsible for fluorescence. Gene expression reporter systems that function under anaerobic conditions have emerged, including the β-glucuronidase gene (*gusA*) (Girbal et al., 2003; Dong et al., 2012; Banerjee et al., 2014), chloramphenicol acetyltransferase gene (*catP*) (Heap et al., 2007; Zhang et al., 2015b), flavin-based fluorescent proteins (Drepper et al., 2007, 2010; Mukherjee and Schroeder, 2015), *luxA-luxB* reporter system (Phillips-Jones, 2000), and *lacZ* gene from *Thermoanaerobacterium thermosulfurogenes* EM1 (Tummala et al., 1999).

### Successful examples of metabolic engineering of gas-fermenting organisms to expand product portfolio

Even though genetic tools and parts for acetogens are still underdeveloped, there are already several successful examples of metabolic engineering of acetogens for non-native product synthesis (Figure 1, Table 2). A first proof of concept study that acetogens can be genetically modified was published in 2010 (Köpke et al., 2010). The study successfully showed the production of non-native butanol in *C. ljungdahlii*. Upon transformation with a plasmid expressing butanol biosynthetic genes (*thlA, hbd, crt, bcd, adhE*, and *bdhA*) from ABE-fermenting *C. acetobutylicum*, the organism was able to synthesize up to 2 mM of butanol from a syngas blend. However, at the end of growth, butanol was assimilated and converted into butyrate. A later study showed improved butanol production from gas in both *C. ljungdahlii* and *C. autoethanogenum* (Köpke and Liew, 2011). A similar biosynthetic operon was used, but under control of a different promoter system and including two electron-transferring flavoproteins, EtfAB, while omitting any external alcohol and aldehyde dehydrogenase genes. The recombinant strains produced butanol as the main product from steel mill gas up to a concentration of 25.7 mM (Köpke and Liew, 2011). Ueki et al. (2014) were able to integrate (via single crossover recombination) the butyrate-biosynthetic pathway (*thlA-crt-bcd-eftB-etfA-hbd-ptb-buk*) from *C. acetobutylicum* by replacing phosphotransacetylase gene *pta*, resulting in the production of ~15 mM butyrate under H_2_/CO_2_ conditions using *C. ljungdahlii*.

**Table 2:** **Summary of genetically modified acetogens**.

**Organism**	**Genetic modifications**	**Results**	**References**
**EXPANDED PRODUCT SPECTRUM**			
*A. woodii*	Plasmid based expression of *C. acetobutylicum* acetone biosynthetic genes (*thlA-ctfAB-adc*) using different combinations of promoter (ackA, pta-ack) and plasmid origin (pIP404, pBP1, pCB102, and pCD6)	Continuous production of 26.4 mg/L/h acetone from synthetic syngas in CSTR	Hoffmeister et al., 2016
*C. aceticum*	Plasmid based expression of *C. acetobutylicum* acetone biosynthetic genes (*thlA-ctfAB-adc*)	Production of up to 9 mg/L acetone from synthetic syngas in bottles	Schiel-Bengelsdorf and Dürre, 2012
*C. aceticum*	Plasmid based expression of synthetic acetone operon of *C. acetobutylicum* thiolase and acetoacetate decarboxylase genes (*thl-adc*) and genes for *B. subtilis* thioesterase (*teII*) or *H. influenzae* acyl-CoA thioesterase (*ybgC*)	Production of up to 59 mg/L acetone a H_2_/CO_2_ gas mix	Becker et al., 2012
*C. autoethanogenum, C. ljungdahlii*	Plasmid based expression of *C. acetobutylicum* butanol biosynthetic genes (*thlA-crt-hbd-bcd*-*etfAB*)	Production of up to 1.93 g/L butanol from steel mill gas and syngas in bottles; Butanol as major product	Köpke and Liew, 2011
*C. autoethanogenum, C. ljungdahlii*	Plasmid based expression of acetone and isopropanol biosynthetic genes *thlA* (from *C. acetobutylicum*) and *ctfA-ctfB-adc* (from *C. beijerinckii*)	Production of up to 300 mg/L acetone and 25 mg/L isopropanol from steel mill gas and syngas in bottles; Continuous production of 700 mg/L/d isopropanol from steel mill gas in CSTR	Köpke et al., 2012
*C. autoethanogenum*	Plasmid based expression of *Chloroflexus aurantiacus* malonyl-coenzyme A reductase	Production of low levels of 3-hydroxypropionate from steel mill gas and H_2_/CO_2_	Köpke and Chen, 2013
*C. autoethanogenum*	Plasmid based expression of *Klebsiella pneumoniae (S)*-specific butanediol dehydrogenase and *Klebsiella oxytoca* diol dehydratase *pddABC* in *C. autoethanogenum* with inactivated native 2,3-butanediol dehydrogenase	Production of up to 370 mg/L *meso*-2,3-butanediol, MEK and 2-butanol from steel mill gas in bottles	Mueller et al., 2013
*C. autoethanogenum*	Plasmid base expression of unspecific acyltransferase from *Acinetobacter baylyi*	Production of low levels of butanoic acid butyl ester from steel mill gas	Liew and Köpke, 2012
*C. autoethanogenum*	Plasmid based over-expression of DOXP synthase, expression of mevalonate pathway, *C. beijerinckii* isopentenyl diphosphate isomerase, and either *Poplar* isoprene synthase or farnesene synthase	Production of low levels of mevalonate, isoprene and franesene from steel mill gas and syngas	Chen et al., 2013a
*C. ljungdahlii*	Plasmid based expression of *C. acetobutylicum* butanol biosynthetic genes (*thlA-crt-hbd-bcd, adhE*-*bdhA*)	Production of up to 148 mg/L *n*-butanol from synthetic syngas in bottles; Conversion to butyrate at end of growth	Köpke et al., 2010
*C. ljungdahlii*	Plasmid expression of *C. acetobutylicum* acetone biosynthetic genes (*thlA-ctfAB-adc*) under lactose inducible promoter	Production of up to 871 mg/L acetone from CO in bottles	Banerjee et al., 2014
*C. ljungdahlii*	Chromosomal integration (single crossover recombination) of *C. acetobutylicum* butyrate biosynthetic genes (*thl*-*crt*-*bcd-etfB*-*etfA*-*hbd*-*ptb*-*buk*) into *pta* promoter region	Production of up to 881 mg/L butyrate under H_2_/CO_2_ in bottles	Ueki et al., 2014
*C. ljungdahlii*	Plasmid based expression of mevalonate pathway, *E. coli* isopentenyl diphosphate isomerase, and *Poplar* isoprene synthase	Production of up to 68 mg/L mevalonate and low levels of isoprene from syngas in bottles	Beck et al., 2014
*C. ljungdahlii*	Plasmid based expression of mevalonate pathway, yeast isopentenyl diphosphate isomerase, and *Poplar* isoprene synthase	Production of up to 68 low levels of isoprene from syngas in bottles	Beck et al., 2014
*M. thermoacetica*	Genome insertion of *ldh* from *Thermoanaerobacter pseudethanolicus* into *pyrF* locus	Heterotrophic production of up to 613 mg/L lactate from glucose in bottles; Autotrophic production not reported	Kita et al., 2013
**ENHANCED PROCESS**			
*A. woodii*	Plasmid based expression of formyl-THF-synthetase, methenyl-THF-cyclohydrolase, methylene-THF-dehydrogenase, and methylene-THF-reductase of *C. ljungdahlii*	Increase in volumetric acetate production rate by 14% under H_2_/CO_2_ conditions in CSTR	Straub et al., 2014
*C. autoethanogenum*	Inactivation of acetolactate decarboxylase gene *budA*	Abolishment of 2,3-butanediol production along with enhanced ethanol production by 79%; Small levels of succinate and lactate produced during growth on steel mill gas	Köpke et al., 2013a
*C. autoethanogenum*	Inactivation of lactate dehydrogenase gene *ldhA*	Abolishment of lactate production	Nagaraju et al., 2015
*C. autoethanogenum*	Plasmid expression of vitamin biosynthetic genes *thiC* of “*C. ragsdalei”* and *panBCD* of *C. beijerinckii*	Successful complementation of thiamine and panthothenate biosynthesis pathways; Strains independent of vitamin B1 and B5 supplementation during growth on steel mill gas	Köpke and Al-Sinawi, 2013
*C. autoethanogenum*	Plasmid expression of native *groES* and *groEL*	Increased cell viability when challenged with ethanol during growth on steel mill gas	Simpson et al., 2011
*C. difficile*	Generation of more than 70,000 unique mutants via transposon mutagenesis, coupled with transposon-directed insertion site sequencing	Identification of 404 essential genes for growth; Identification of 798 genes that are likely to affect sporulation	Dembek et al., 2015
*C. ljungdahlii*	Chromosomal deletion of *adhE1* and/or *adhE2*	6-fold reduction in ethanol concentration of Δ*adhE1* mutant (but not Δ*adhE2* mutant) under heterotrophic conditions	Leang et al., 2013
*C. ljungdahlii*	Plasmid expression of formate dehydrogenase from *E. coli*	4.3-fold increase in intracellular NADH concentration; 2.3-fold improvement in maximum power density in a sodium formate fed microbial fuel cell	Han et al., 2016

Besides butanol and ethanol, production of the other solvents of ABE/IBE fermentation have been demonstrated from gas. By delivering a plasmid that expresses an acetone operon (*ctfA, ctfB, adc*, and *thlA*) of *C. acetobutylicum* under the control of the *thlA* promoter, *C. aceticum* was demonstrated to produce up to 0.14 mM acetone using H_2_/CO_2_ gas (Schiel-Bengelsdorf and Dürre, 2012). Acetone production increased over 500x from H_2_/CO_2_ in *C. ljungdahlii* using the same genes but under control of an inducible system (Banerjee et al., 2014) and in *A. woodii* by combinatorial testing of different promoter and plasmid combinations (Hoffmeister et al., 2016). In a different study, it was shown that acetone, depending on the redox state, could be converted to isopropanol in both *C. ljungdahlii* and *C. autoethanogenum* (Köpke et al., 2012). If acetone is targeted for production, it is however desirable to either use an organism like *A. woodii* that is unable to reduce acetone to isopropanol or eliminate this function if the host is *C. autoethanogenum* or *C. ljungdahlii* (Hoffmeister et al., 2016). An NADPH dependent primary:secondary alcohol dehydrogenase capable of catalyzing the reduction of acetone to isopropanol was identified to be present in these two organisms (Köpke et al., 2014) and inactivation of this single gene indeed renders the organism unable to reduce acetone further (Köpke et al., 2015). Rather than using genes from ABE fermentation organisms, acetone production was also demonstrated via a thioesterase *teII* of *Bacillus subtilis* and an acyl-CoA thioesterase *ybgC* of *Haemophilus influenzae*, which improved acetone production in *C. aceticum* from 0.24 mM to 1 mM on a H_2_/CO_2_ gas mix (Becker et al., 2012; Schiel-Bengelsdorf and Dürre, 2012).

Another example is heterologous production of lactate in *M. thermoacetica*. The gene *ldh* (encodes lactate dehydrogenase) from *Thermoanaerobacter pseudethanolicus* was inserted into the genome, resulting in the production of 6.8 mM lactate. Experiments were, however, performed on fructose rather than gas (Kita et al., 2013). Even energy-intense products like isoprene, which require several molecules of ATP, have been successfully produced in acetogens growing on gas. Multiple groups have demonstrated a proof of concept for isoprene production by installation of the mevalonate pathway and an isoprene synthase in either *C. autoethanogenum* or *C. ljungdahlii* (Beck et al., 2013, 2014; Chen et al., 2013a; Furutani et al., 2013). In order to optimize these processes, it is important to understand the energy and redox metabolism of acetogens, as this is significantly different to sugar fermenting organisms and governs the product profile (Marcellin et al., 2016).

LanzaTech has demonstrated production of several additional high value molecules via gas fermentation, such as methyl ethyl ketone (MEK) (Mueller et al., 2013), 3-hydroxypropionate (3-HP) (Köpke and Chen, 2013), biodiesel, and jet fuel molecules (Liew and Köpke, 2012). The company also works together with the world's largest nylon producer, U.S.-based Invista, on new processes for production of nylon precursor 1,3-butadiene (Köpke and Havill, 2014; INVISTA, 2015), French start-up Global Bioenergies on direct isobutylene production (Global Bioenergies, 2016), and the German company Evonik Industries on a new process for production of specialty plastics from gas (Evonik, 2013; LanzaTech, 2013).

In addition to expand the product spectrum of acetogens, significant work has also been carried out to enhance current processes (Table 2). Work has focused on improving substrate utilization and ethanol production in the gas fermentation process and eliminating the need to supplement the microbe with vitamins to reduce costs.

Substrate utilization could be improved by overexpressing the WLP. Straub et al. overexpressed the four THF-dependent enzymes of the methyl branch of *C. ljungdahlii* in *A. woodii*, resulting in 14% increase in volumetric acetate production rate (Straub et al., 2014). In a similar attempt, formate dehydrogenase (Fdh) of *E. coli* was heterologously expressed in *C. ljungdahlii* facilitate electron transfer in electrosynhesis. It was shown that the intracellular NADH concentration increased by 4.3-fold leading to a process improvement (Han et al., 2016).

To improve ethanol production, competing pathways such as the butanediol production pathway were inactivated in *C. autoethanogenum*. This was achieved by inactivation of the *budA* gene (encoding an acetolactate decarboxylase) using either allelic exchange or ClosTron mutagenesis (Köpke et al., 2013a). During growth on steel mill gas, the *budA* deficient mutant synthesized no 2,3-butanediol, but generated 79% more ethanol and 9.9-fold higher lactate than the wild-type control. Interestingly, also significant amount of platform chemical succinate were detected in this engineered strain as an overflow metabolite from the incomplete TCA cycle. A similar effect has also been observed by inactivating the lactate dehydrogenase (*ldhA*) of the same strain (Nagaraju et al., 2015). To alleviate product inhibition of ethanol, native chaperones GroES and GroEL were overexpressed in *C. autoethanogenum* with the aim of protecting proteins from misfolding (Simpson et al., 2011). When challenged with various amounts of ethanol [1.5–6% (w/v)], the chaperone overexpression strain consistently outperformed the control strain during growth on steel mill gas. While not resulting in an improvement for ethanol production, important insight came from two studies in *C. ljungdahlii*, where the role of ethanol biosynthesis genes *adhE1* and/or *adhE2* was studied and confirmed by generated chromosomal deletions and doing complementation experiments (Leang et al., 2013; Banerjee et al., 2014).

As supplementation of vitamin represent a significant cost factor in a process, work has also been carried out to eliminate the need for vitamin supplementation. An *in silico* analysis showed that thiamine (vitamin B_1_) and panthothenate (vitamin B_5_) biosynthetic pathways in *C. autoethanogenum* are incomplete but only lack a few genes. A complementation of both pathways was demonstrated by heterologously expressing *thiC* of “*C. ragsdalei”* and *panBCD* of *C. beijerinckii* in *C. autoethanogenum*. It was not only shown that the resulting strains no longer need supplementation with these vitamins but *thiC* and *panBCD* genes could also be used as selective markers (Köpke and Al-Sinawi, 2013).

### Installation of wood-ljungdahl pathway in non-acetogens

Given the challenges working with acetogens (Burk and Van Dien, 2016), it has also been attempted to install the WLP in traditional hosts as *E. coli* or yeast.

First attempts to demonstrate parts of the WLP in *E. coli* were already carried out in 1989. Genes encoding methyltransferase (MeTr), CODH/ACS, and CoFeSP from *M. thermoacetica* were cloned and heterologously expressed in *E. coli* (Roberts et al., 1989). Although the MeTr was found to be active, activity was not detected for the other two enzymes, and both CODH and CoFeSP were found to be less thermostable than when isolated from the native host. In a later study, it could be shown that the CODH/ACS was functional in both CO oxidation and CO/acetyl-CoA exchange activities after heterologous expression in and purification from *E. coli* (Loke et al., 2000).

With the advancements in strain engineering, it became possible to express the complete WLP from various source organisms in *E. coli* (Burk et al., 2010; Trawick et al., 2012). While certain aspects could be demonstrated, growth on CO and H_2_ could not be shown. One challenge for successful operation of the WLP in non-acetogenic hosts is a lack of intracellular conditions and genetic outfit of traditional hosts as *E. coli* or yeast promoting co-factor production and assembly of delicate metal centers. Therefore, in an alternative approach, it has also been attempted to implement the pathway into more closely related ABE-fermenting model organism *C. acetobutylicum* (Al-Hinai et al., 2012a,b). While heterologous gene expression was demonstrated using qRT-PCR and some enzymatic activities were shown, the resultant recombinant *C. acetobutylicum* was unable to grow autotrophically. The other challenge to make this work may also be yet unknown genes that play a function in operation of the WLP. For methanogens for example it has been predicted that over 200 genes are involved in growth on CO_2_/H_2_ (Kaster et al., 2011).

Though a promising approach in theory (in particular in light of the advancements to design and generate synthetic genomes Hutchison et al., 2016), the most efficient use of the WLP in the near future is likely to remain in acetogens.

## Coupled processes to expand product spectrum of gas fermentation

While synthetic biology approaches offer great potential to expand the product spectrum of acetogens, coupling gas fermentation with other biological or chemical platforms can allow for generation of products currently impossible to synthesize or economically unviable with gas fermentation alone. The following sections describe specific examples of coupled processes involving gas-fermenting microbes that have the ability to expand the gas fermentation product portfolio and improve product yields and economics. Coupled processes include multi-step bioprocesses (Section Coupling to Other Bioprocesses) that convert metabolites from a primary fermentation in a secondary fermentation (Agler et al., 2011) as well as using heterogeneous catalysts (Section Coupled Catalytic Processes) to chemically convert gas fermentation products.

### Coupling to other bioprocesses

Multi-step bioprocesses can consist of combinations of pure cultures, co-cultures, and/or open (undefined) mixed cultures with natural and/or genetically modified strains. Potential advantages of co-cultures and mixed cultures include microbial symbiotic relationships, increased substrate utilization, and improved product yields. For example, undesirable inhibitory or toxic byproducts of a metabolic pathway can be consumed by a second microbe, or two host cells can work together to ensure the optimal environment for all pathway enzymes by effectively dividing the pathway (Zhang et al., 2015a; Zhou et al., 2015). Other advantages of mixed cultures include protection against contamination by foreign bacteria, resistance to bacteriophages, oxygen removal and production of growth factors by facultative anaerobes in an anaerobic reactor (Angenent and Wrenn, 2008), improved stability, and obligately mutual metabolic activity (syntrophy) that enables production or degradation of complex organics (Morris et al., 2013). As compared to pure cultures, disadvantages include potentially longer period to reach steady-state, difficulty understanding the interaction between microbes in the fermentation, reduced reproducibility due to challenges in maintaining population dynamics, and inability to optimize parameters for each strain.

#### H_2_/CO_2_ to lipids

Most gas fermentation work to date has been carried out on CO-containing gas streams. CO acts as both a carbon and energy source, providing sufficient energy to synthesize reduced products such as ethanol and butanol. Using CO_2_ as the carbon source for ethanol and other reduced product formation is highly desirable but difficult to achieve. The more oxidized CO_2_ can only act as a carbon source and typically requires H_2_ for its fixation. H_2_/CO_2_ gas fermentations mostly produce acetate, though production of ethanol (Mock et al., 2015) and other products such as acetone (Schiel-Bengelsdorf and Dürre, 2012; Hoffmeister et al., 2016) or butyrate (Ueki et al., 2014) have been described, albeit at lower productivity compared to CO. Additionally, ethanol production from CO is thermodynamically favorable over CO_2_ (Reactions 1-4; the free energy (ΔG°') of Reactions 1–4 was calculated from data given in Thauer et al. (1977)).

(1) $$\text{6 CO + 3 }{\text{H}}_{2}\text{O }⇌\;{\text{C}}_{2}{\text{H}}_{5}\text{OH + 4 }{\text{CO}}_{2}\;\Delta {\text{G}}^{{\text{o}}^{\prime }}=-217\text{ kJ/mol}$$

(2) $$\text{3 CO + 3 }{\text{H}}_{2}\;⇌\;{\text{C}}_{2}{\text{H}}_{5}\text{OH + }{\text{CO}}_{2}\;\Delta {\text{G}}^{{\text{o}}^{\prime }}=-156.9\text{ kJ/mol }$$

(3) $$\text{2 CO + 4 }{\text{H}}_{2}\;⇌{\text{C}}_{2}{\text{H}}_{5}\text{OH + }{\text{H}}_{2}\text{O }\Delta {\text{G}}^{{\text{o}}^{\prime }}=-136.8\text{ kJ/mol }$$

(4) $$\text{2 }{\text{CO}}_{2}\text{ + 6 }{\text{H}}_{2}\;⇌{\text{C}}_{2}{\text{H}}_{5}\text{OH + 3 }{\text{H}}_{2}\text{O }\Delta {\text{G}}^{{\text{o}}^{\prime }}=-96.7\text{ kJ/mol}$$

Using CO as the sole carbon and energy source to form ethanol results in CO_2_ formation (Reaction 1), lowering the amount of fixed carbon. The presence of H_2_ can reduce or eliminate CO_2_ (Reactions 2–4), but in practice, the H_2_ uptake is often limited in the presence of CO through hydrogenase inhibition by CO (Purec et al., 1962; Bennett et al., 2000; Pandelia et al., 2010). To obtain the desired H_2_ uptake to limit CO_2_ production, the dissolved CO concentration must therefore be low. Unfortunately, under such conditions, organic acid (e.g., acetate and butyrate) production becomes the favored fermentation product over more reduced alcohols (e.g., ethanol, butanol) (Hurst and Lewis, 2010; Devarapalli and Atiyeh, 2015). Using CO_2_ as the carbon source does not inhibit H_2_ uptake as seen with CO but again results in lower productivity and is thermodynamically less favorable than using CO due to the lower energy content of CO_2_.

If acetate, on the other hand, is the target of the fermentation, using H_2_/CO_2_ is an attractive option. It has been demonstrated that *A. woodii*, for example, can be used for productive fermentations, with a space time yield of 148 g/L/d of acetate in a bioreactor with a submersed filtration unit and a final acetate concentration of 44 g/L after 11 days (Kantzow et al., 2015). Even though acetate can be produced at high volumetric production rates, the process has some challenges that need to be overcome to make it economically viable. The fermentation process requires the costly constant addition of base to compensate for the acid produced in order to keep the pH constant. Post fermentation recovery of the acetate then requires the addition of another strong acid to protonate the acetate.

To overcome economic drawbacks of acetate fermentation, a secondary fermentation process can be employed wherein acetate is upgraded to more valuable products. For instance, Gong et al. demonstrated lipid synthesis from acetate by multiple oleaginous yeast strains with one, *Cryptococcus curvatus* ATCC 20509, yielding a lipid concentration of 4.2 g/L and 73.4% of the cell dry weight (Gong et al., 2015). The acetate in this work was not sourced from gas fermentation, however. Recent work from Hu et al., presented an integrated bioprocess where acetate made from CO_2_ and H_2_ by *M. thermoacetica* was converted to lipids by *Yarrowia lipolytica* (Hu et al., 2016). The two-stage system produced 18 g/L of triacylglycerides at 36% lipid content of cell weight. This work not only validates this coupled bioprocess approach but also provides a baseline for further experiments. One potential improvement, for example, could be made in the efficiency of carbon utilization by feeding any CO_2_ produced by the yeast back to the acetogen (Stephanopoulos, 2011).

#### Gas to elongated carboxylic acids and conversion to alcohols

Longer-chain linear alcohols than ethanol are more valuable as drop-in fuels due to their energy density, compatibility with current transportation infrastructure, and greater ease of separation from aqueous fermentation broth. They are also valuable commodity chemicals, as production of linear alcohols through petrochemical means can be challenging since existing technologies such as linear alpha oligomerization are not selective. Gas-fermentation products of acetogens can be converted to longer carboxylic acids by other microorganisms, and a number of microorganisms, including acetogens themselves, can reduce carboxylic acids of varying lengths to their corresponding alcohols. Coupling these processes could then allow for synthesis of low-carbon drop-in fuels or valuable chemicals from gas feedstocks.

Chain elongation of short-chain fatty acids and alcohols such as acetate and ethanol to longer carboxylic acids can occur via the reverse β-oxidation pathway (Barker et al., 1945; Spirito et al., 2014; Angenent et al., 2016). Two molecules of acetyl-CoA (sourced from ethanol) condense to form acetoacetyl-CoA, which is then reduced to butyryl-CoA. The energy required for elongation and subsequent reduction can be provided indirectly by the oxidation of ethanol to acetyl-CoA (Spirito et al., 2014), which provides reducing equivalents such as NADH and ATP through SLP and the Rnf complex as described above for acetogens. As this is a cyclical pathway, multiple rounds of elongation can take place with the addition of acetyl-CoA with each cycle. Use of this approach has resulted in production of caproate (C6) and caprylate (C8) from acetate with either ethanol or H_2_ as the electron donor source (Steinbusch et al., 2011; Angenent et al., 2016). Chain extension for longer odd-numbered carboxylic acids can also occur if propionate (C3) is provided as the substrate, which is converted to valerate (C5) and eventually heptanoate (C7) (Grootscholten et al., 2013).

This approach has already shown to successfully couple to acetogenic gas fermentation. A dilute ethanol and acetate product stream from syngas fermentation with *C. ljungdahlii* was used as the substrate for a separate anaerobic reactor, which produced butyrate and caproate (Vasudevan et al., 2014). Because mixed cultures used for chain elongation may also convert substrates such as acetate to methane by acetoclastic methanogens, care must be taken to ensure culture conditions do not favor methanogen growth, which would limit the yield of longer chain carboxylic acids. Techniques to inhibit acetoclastic methanogens include pH control (Agler et al., 2014) and high H_2_ partial pressure, which is estimated to confer greater growth advantage to homoacetogens over hydrogenotrophic methanogens (Spirito et al., 2014).

Despite the prevalent use of mixed cultures, these works and others allow for speculation that the non-acetogenic *C. kluyveri* may be responsible for the chain elongation based on previous characterizations of that microorganism (Barker et al., 1945; Seedorf et al., 2008). *C. kluyveri* was the only anaerobic, clostridial bacterium isolated to pure culture known to utilize ethanol and acetate as sole energy sources until recently with the isolation of “*Clostridium pharus”* (Levinson, 2014). Because *C. kluyveri* is considered the dominant chain-elongating microorganism in some of these cases, it could be possible to couple a pure culture of this bacteria to a gas fermentation platform to achieve longer chain carboxylic acids from syngas.

Gas-fermenting acetogens can be used to reduce carboxylic acids into their respective alcohols with CO or H_2_ present in syngas as a source of electron donors (Steinbusch et al., 2008; Simpson et al., 2009a). The carboxylic acid is reduced to its corresponding aldehyde *via* the reduced ferredoxin-dependent AOR (Köpke et al., 2010; Isom et al., 2015; Mock et al., 2015). The reduction of the aldehyde to the corresponding alcohol is completed by an alcohol dehydrogenase. Pure cultures of *C. ljungdahlii* and “*C. ragsdalei”* have been reported to convert externally added carboxylic acids (acetate, propionate, *n*-butyrate, iso-butyrate, *n*-valerate, and *n*-caproate) into their corresponding alcohols using syngas as the source of electrons (Perez et al., 2013; Isom et al., 2015). Isom et al. for example, showed “*C. ragsdalei”* can convert caproate at 62%, valerate at 82%, and butyrate at 100% efficiency to their respective alcohols.

This capability possessed by acetogens allows for interesting coupling scenarios to produce alcohols that were chain extended from ethanol and acetate. Coskata, for example, had described using a symbiotic co-culture of C1-fixing and C3-producing microorganisms or, alternatively, a multi-zone process containing these microorganisms in separate zones. Syngas would be consumed and converted to ethanol and acetate by acetogens in one fermentation zone (Datta et al., 2014; Enzien et al., 2014). These products would transfer to the second fermentation zone where they could be converted to propionate by separate microorganisms. This could then be reduced to propanol using acetogens and syngas as an electron donor back in the first fermentation zone. A similar syntrophic co-culture of *C. kluyveri*, an acetogenic clostridium, and/or a butyrogenic microorganism for the production of butanol was also described by Coskata (Datta and Reeves, 2014). Here the acetogen would produce ethanol and acetate, which could be converted to butyric acid and butanol by whichever species contain the genes for NADPH dependent acetyl-CoA reductase and either a butyryl-CoA acetate transferase or a butyrate kinase. Diender et al. recently demonstrated production of butyrate, caproate, butanol and hexanol by co-culture of *C. kluyveri* and *C. autoethanogenum* fed with CO and H_2_ (Diender et al., 2016).

Richter et al. even integrated three bioprocesses to produce alcohols, with the final step of carboxylic acid conversion to alcohols completed by syngas fermentation (Richter et al., 2013a). First, the conventional yeast fermentation product corn beer was fed to an undefined anaerobic culture where butyrate and caproate were produced. The caproate product was selectively removed and the effluent from this process was then fed to the acetogen *C. ljungdahlii*, which reduced the residual short and medium chain carboxylic acids (*n*-butyric acid and unremoved *n*-caproic acid) into their respective alcohols using syngas as an electron source.

### Coupled catalytic processes

Another option for expanding the products formed from gas fermentation is coupling gas fermentation to chemical synthesis platforms. Combining biologically synthesized products from gas fermentation with conversion processes employing heterogeneous catalysts may allow for conversion of syngas substrates to products that may be difficult or currently impossible to produce solely via enzymatic catalysis (e.g., diesel/jet fuels and thermoplastic polymers). These chemical processes are generally faster than biological syntheses, and, if performed carefully, may still offer an overall reduction in GHG emissions for these products compared to traditional production from fossil fuels. Two reviews published recently focus on coupling biological fermentation products with chemical processes and explore more in depth the scope of work accomplished to date (Goulas and Toste, 2016; Schwartz et al., 2016). Detailed here are processes more immediately relevant to commercial gas fermentation efforts and/or not covered in those reviews.

#### Alcohol-to-jet fuel

Although ethanol (and potentially other short-chain alcohols) produced by gas fermentation can serve as an additive or potential replacement to gasoline, it is not considered to be an energy dense enough molecule to serve as a jet fuel. Jet fuels are typically aliphatic and contain 8–16 carbon atoms (Atsonios et al., 2015). Molecules this large can be made from syngas substrates via coupled biological processes (see above), but it may be more efficient to convert the small alcohols to jet fuel chemically by employing a process called alcohol-to-jet (ATJ).

To obtain jet fuel from these small alcohols, ethanol and butanol (separated from the fermentation broth) are first dehydrated to corresponding alkenes ethylene and butylene (Harvey and Meylemans, 2011; Zhang and Yu, 2013). The alkenes are then oligomerized to a desired range of sizes (i.e., number of carbon atoms) that fit the end application (Heveling et al., 1998; Harvey and Meylemans, 2011). Reaction conditions are tuned to avoid extensive polymerization to thermoplastics like poly(ethylene) when fuels are the desired product (Atsonios et al., 2015).

There are currently multiple industrial ATJ projects for both ethanol and butanol conversions to jet fuel (Lane, 2015a; LanzaTech, 2015). The ATJ fuels have undergone and continue to undergo rigorous testing to establish their use as drop-in fuels for commercial aircraft (Luning Prak et al., 2015). Successfully meeting these standards will expand the volume of fuels that may be sourced from renewable resources, such as biomass, and aid in keeping fossil fuels typically needed for aviation fuel in the ground.

Another promising approach to convert ethanol (and other short-chain alcohols) to jet (or biodiesel) fuels is via extractive catalytic upgrading directly from the fermentation broth (Anbarasan et al., 2012; Bormann et al., 2014; Sreekumar et al., 2014, 2015; Baer et al., 2016). This technology requires strains that co-produce acetone (Becker et al., 2012; Köpke et al., 2012, 2015; Schiel-Bengelsdorf and Dürre, 2012) which harbors a nucleophilic α-carbon amenable to C–C bond formation with the electrophilic alcohols.

#### Ethanol and butanediol to butadiene

As mentioned, alkenes derived from gas fermentation alcohols are sources for ubiquitous thermoplastic and rubber products. Ethylene made from dehydration of ethanol can be more fully polymerized beyond oligomers for ATJ fuel and converted to poly(ethylene). Poly(butadiene) is another polymer used in mass production of products such as nylon or automobile tires. The monomer of this synthetic rubber, 1,3-butadiene, can be formed from gas fermentation products ethanol (Makshina et al., 2012) and 2,3-butanediol (Duan et al., 2015). These processes have been reviewed more in depth elsewhere (Makshina et al., 2014). 1,3-butadiene is also a precursor for nylon production (Carraher, 2010), and LanzaTech is currently a part of multiple collaborations to develop gas fermentation production of 2,3-butanediol for the purpose of chemical conversion to 1,3-butadiene (Köpke and Havill, 2014).

## Summary and outlook

Gas fermentation has developed incredibly over the past years, leading to multiple endeavors for commercial production of ethanol from syngas. Significant strides in genetic manipulation of gas-fermenting bacteria (acetogens specifically) has granted access to new products and routes for enhancing production rates and yields. A focus for the future is continuing to develop these genetic tools and techniques and move toward high-throughput genetic engineering and screening. Given the challenges inherent in heterologous pathway expression (e.g., balancing of fluxes of each enzyme reaction to reduce accumulation of toxic intermediates, redox imbalance, and metabolic burden from overproduction of enzymes), an automated platform would allow for larger-scale and greater efficiency in testing modifications.

While these tools and platforms develop, coupled processes, both biological and chemical, presently allow for synthesis of products currently unattainable in single gas-fermenting microorganisms. However, the urgency of global climate change hangs over this work. Even if these advances in gas fermentation are able to expand to produce aviation fuels and thermoplastics at commercially-relevant rates, it is unlikely to develop quickly enough to completely replace the consumption of fossil fuels for their production. The combination of gas fermentation with established chemical processes, however, could more quickly bring renewable aviation fuels and synthetic rubber to relevancy. Sourcing fuels and chemicals for polymer synthesis from renewable resources like biomass may be attainable with gas fermentation.

## Author contributions

FL, MM, RT, CM, and MK drafted the manuscript and all authors edited and wrote the manuscript. FL, MM, RT contributed equally. All authors gave approval for publication of the manuscript.

## Funding

We thank the following investors in LanzaTech's technology: Sir Stephen Tindall, Khosla Ventures, Qiming Venture Partners, Softbank China, the Malaysian Life Sciences Capital Fund, Mitsui, Primetals, CICC Growth Capital Fund I, L.P. and the New Zealand Superannuation Fund.

### Conflict of interest statement

The authors declare that the research was conducted in the absence of any commercial or financial relationships that could be construed as a potential conflict of interest. LanzaTech, Inc. has commercial interest in gas fermentation.
